# An integrated nutritional and performance monitoring framework for athletes with celiac disease: A structured narrative review

**DOI:** 10.1016/j.crfs.2026.101568

**Published:** 2026-09-22

**Authors:** Osaid B. Yamani, Maha M. Alghamdi, Alyaa M. Zagzoog, Essra A. Noorwali, Rahaf A. Alotaibi, Abdullah F. Alghannam, Khalid S. Aljaloud, Alan A. Aragon, Nour A. Elsahoryi

**Affiliations:** a Sports Strategy Planning & Governance, The Royal Commission for AlUla, AlUla, Saudi Arabia; b Department of Clinical Nutrition, College of Applied Medical Sciences, Umm Al-Qura University, Makkah, Saudi Arabia; c Department of Community Health, Faculty of Applied Medical Sciences, Northern Border University, P.O. Box 715, Arar, 91431, Saudi Arabia; d Clinical Nutrition Department, Faculty of Applied Medical Sciences, Umm Al-Qura University, Makkah, Saudi Arabia; e Department of Community Health Sciences, College of Applied Medical Sciences, King Saud University, Riyadh, Saudi Arabia; f Lifestyle and Health Research Center, Natural and Health Sciences Research Center, Princess Nourah Bint Abdulrahman University, P.O. Box 84428, Riyadh, 11671, Saudi Arabia; g Department of Exercise Physiology, College of Sport Sciences and Physical Activity, King Saud University, P. O. Box 2454, Riyadh, 11451, Saudi Arabia; h Fit Advancement, LLC. Chatsworth, California, USA; i Department of Nutrition, Faculty of Pharmacy and Medical Sciences, University of Petra, Amman, Jordan

**Keywords:** Celiac disease, Athletes, Sports nutrition, Gluten-free diet, Performance monitoring, Micronutrient deficiency

## Abstract

Celiac disease (CD) is an important yet under-researched condition among athletes, with the potential to disrupt nutritional strategy, recovery, and performance. The purpose of this structured narrative review is to synthesize the current evidence on nutrient deficiencies, dietary management, and performance-monitoring strategies specific to athletes with CD, and to propose an integrated framework for their nutritional and functional assessment. This review was conducted according to the Scale for the Assessment of Narrative Review Articles (SANRA) and compiled from literature published in PubMed/MEDLINE, Cochrane, BioMed Central, and the BMJ collection up to 2025, and included the following: global prevalence estimates, genetic susceptibility pathways, dietary management of the Gluten-Free Diet (GFD), and monitoring of micronutrients and functional performance. While maintaining a GFD is key to avoiding intestinal damage and to health, it is also challenging given the limitations of the diet and potential for nutritional deficiency. CD has a complex, multi-faceted impact on athletes, including both physiological (iron, vitamin D, calcium, magnesium, zinc, B-vitamin deficiencies) and neurological (systemic, low-grade inflammation, altered gut–brain axis signalling) and psychological (high odds of anxiety (OR: 6.03) and depression (OR: 2.17) compared to healthy controls and social isolation due to dietary restrictions). Comprehensive dietary planning and continual nutritional monitoring are essential to good management.

The review highlights specific gaps in awareness among coaches, athletic trainers, and sports dietitians regarding gluten cross-contamination risk, timing-sensitive micronutrient monitoring, and recognition of disordered eating patterns in athletes with CD, and calls for structured CD education within sports-medicine and sports-nutrition curricula. It concludes by identifying three priority research directions: adequately powered, biopsy-confirmed trials stratified by sport type, sex, and mucosal healing status; mechanistic studies of exercise-induced intestinal permeability using biomarkers such as zonulin and I-FABP; and development of a validated athlete-specific CD screening tool alongside an international performance-monitoring registry.

## Highlights

• CD athletes face nutrient malabsorption, psychosocial burden, and GFD challenges.
• Personalized nutrition and micronutrient monitoring are critical for CD athletes.
• Iron, vitamin D, calcium, and B12 deficiencies require routine assessment in CD.
• Effective monitoring integrates dietary, clinical, biochemical, and functional markers.
• Multidisciplinary collaboration enhances health and performance in CD athletes.

## Graphical abstract

**Figure:**
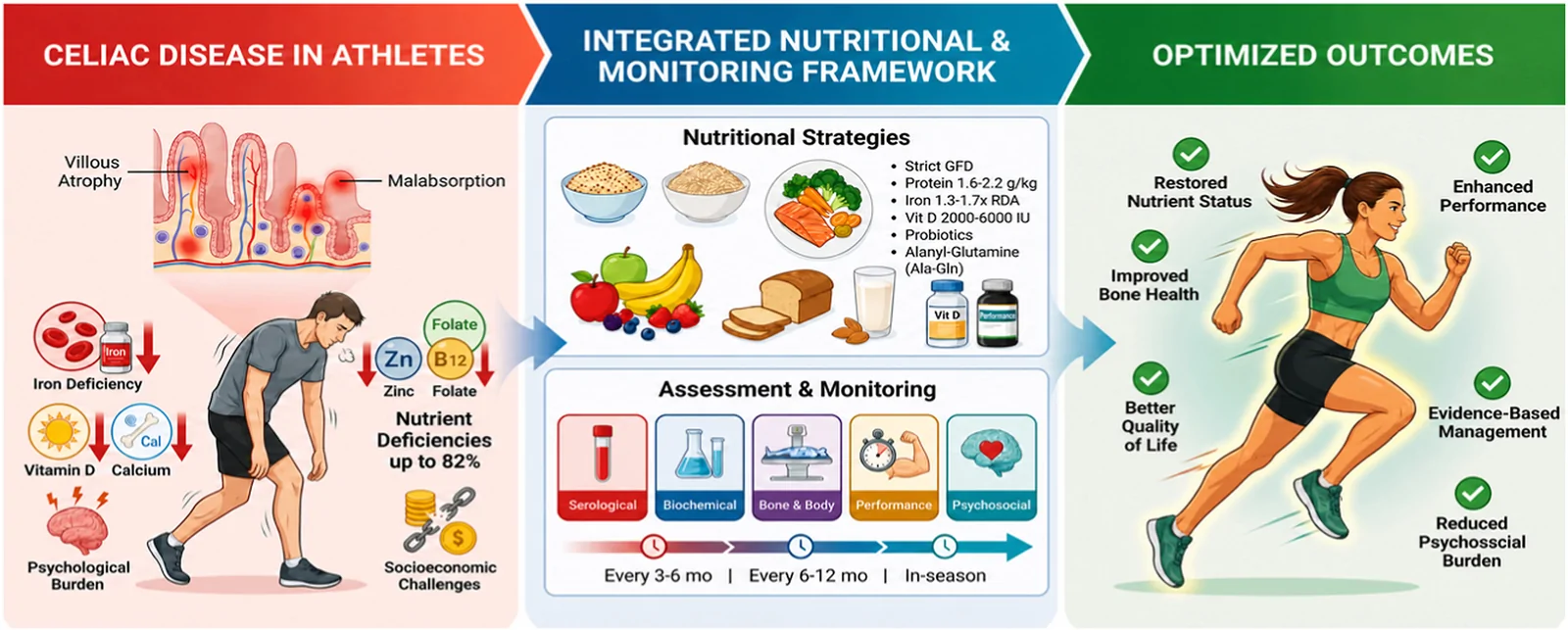

## 1 Introduction

Dietary gluten triggers an adaptive immune response in genetically susceptible individuals, resulting in progressive mucosal injury with villous atrophy, crypt hyperplasia, and intraepithelial lymphocytosis, which in turn decreases the absorptive surface area and compromises the integrity of the epithelial barrier, and has far-reaching implications for general health (Rodrigo, 2006; Elsahoryi., 2021) (Fig. 1), leading to nutrient deficiencies and a wide range of health consequences. Deamidation of gliadin peptides by tissue transglutaminase-2 and the Human Leukocyte Antigen (HLA)-DQ2/DQ8-restricted T-cell immune activation leads to the pathogenesis that culminates in villous atrophy and crypt hyperplasia (Rubio-Tapia et al., 2023). Clinically, CD is confirmed through a combination of positive serology (tissue transglutaminase and endomysial antibodies) and small-intestinal biopsy demonstrating villous atrophy, which remains the diagnostic reference standard (Rubio-Tapia et al., 2023); this distinction is clinically important because many individuals who adopt a gluten-free diet or report gluten-related symptoms have never undergone biopsy-based confirmation. Based on serological screening data, the prevalence of Celiac Disease (CD) is estimated to be around 1.4% of the global population, but the actual prevalence is significantly underestimated due to the 'iceberg' effect of CD (Singh et al., 2018; Catassi et al., 2014). There are limited prevalence data for athletes, and those data mostly rely on self-report methods only, with no serological or histological confirmation, also making it difficult to make definitive estimates for comparison (Leone et al., 2020).

**Fig. 1:**
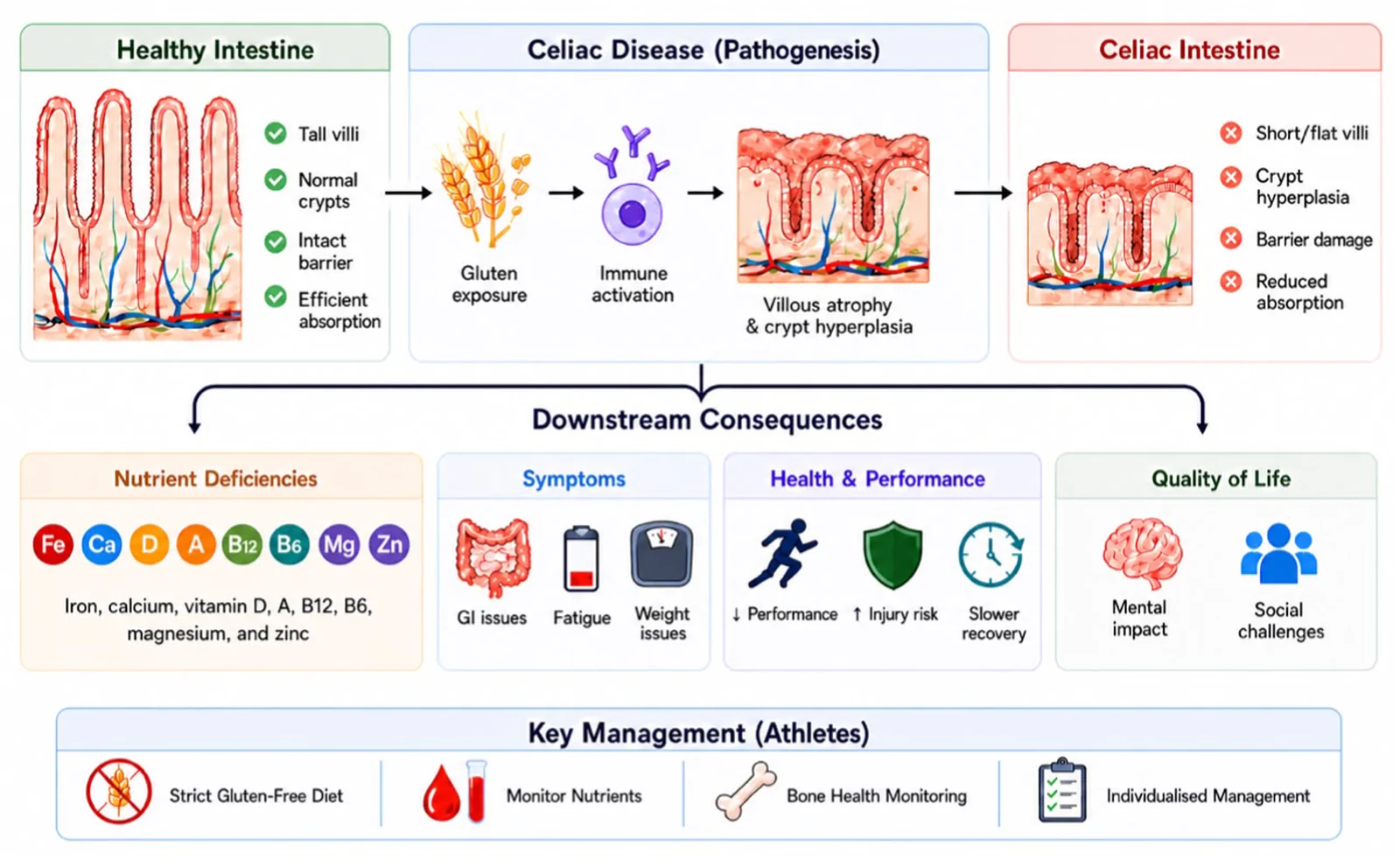
Intestinal villous atrophy in CD relative to healthy mucosa, with downstream consequences for nutrient absorption, mucosal barrier integrity, and systemic health.

Remarkably, the iceberg model suggests that only a small fraction of the burden of disease is clinically manifest, and large numbers of individuals with the disease will be clinically unmanifest, without gastrointestinal cancer, due to a nonclassical presentation. A lack of specificity is also present in athletes, as gastrointestinal symptoms in these individuals can be attributed to both CD and exercise-induced intestinal dysfunction, with passive case-finding strategies being inadequate (Catassi et al., 2014; Costa et al., 2017). However, there are no good athlete-specific prevalence data; the recent reviews on burden of disease and challenges of management in athletes with CD are based on an increased prevalence of CD among athletes and not the burden of disease in athletes (Leone et al., 2020). Due to the large proportion of undiagnosed CD, however, the true incidence of the disease is difficult to assess (Kvamme et al., 2022). CD manifestation varies widely across ethnic groups and geographic locations and can occur at any stage of the human life cycle (Singh et al., 2018).

Pathophysiological consequences of CD involve impaired absorption of essential nutrients such as iron, calcium, magnesium, zinc, vitamin B12, folate, vitamin D, and protein (Montoro-Huguet et al., 2021; Ganji et al., 2019; Casella et al., 2016), which can lead to various clinical complications, including (but not limited to) anemia, osteoporosis, and neurological imbalances. This malabsorptive state can lead to decreased performance, slower recovery rates, and increased injury and illness prevalence for athletes (Ghazzawi et al., 2023). Moreover, people with CD often have gastrointestinal symptoms such as abdominal pain, diarrhoea and abdominal distention (Caio et al., 2019). Beyond the home environment, multiple studies have documented that foods labeled gluten-free (GF) frequently contain detectable gluten, posing risks during travel and competition (Lerner et al., 2019). The gastrointestinal symptoms, along with malabsorption, have a significant effect on Quality of Life (QOL) and the psychological health of athletes who have the condition (Leone et al., 2020; Harnett and Myers, 2020).

Patients with CD should be managed by following a strict Gluten-Free Diet (GFD) regimen (Al-Toma et al., 2019). The strictness of this dietary regimen is key in reducing intestinal mucosal damage and alleviating its negative health effects (Al-Toma et al., 2019). However, these athletes face a great challenge in reconciling the strict dietary requirements of sports activities with the limitations of the GFD (Kreutz et al., 2020; D'angelo et al., 2020; Cialdella-Kam et al., 2016; Shepherd et al., 2017; Vici et al., 2016; Cardo et al., 2021). These can be exacerbated by the lower nutritional content of GF products (Miranda et al., 2014; Calvo-Lerma et al., 2019). The Food and Drug Administration (FDA) has established a criterion for GF as <20 ppm gluten in the U.S. and has extended its criteria to fermented/hydrolyzed foods, if applicable to sports foods and drinks (Hahn, 2020). For athletes suffering from CD, it is important to make sure they are eating a well-rounded and diverse diet to get sufficient macronutrients and micronutrients to support their performance (Thomas et al., 2016). People with CD need to navigate a complicated nutritional world, which requires careful planning of meals, careful reading of product labels, and a thorough understanding of safe food choices – and an understanding of where gluten may be found in unexpected foods. (Gutowski et al., 2020).

The effects of CD do not just occur physically and can venture into psychological and social areas of those impacted athletes, which can lead to social anxiety and depression (Lee et al., 2021; Clappison et al., 2020). It can be challenging to explain the dietary needs to coaches, teammates, and event organizers, and may require extra efforts to ensure that special food needs are met in these settings. In order to handle these complexities, it is crucial to adopt a holistic nutritional management approach guided by systematic monitoring and assessment of athletes with CD (Cheng and Handu, 2020). The proper and timely detection and correction of nutritional deficiencies in this population requires careful monitoring of serum levels of iron indices (ferritin, transferrin saturation, reticulocyte haemoglobin content), 25-hydroxyvitamin D, calcium, magnesium, zinc, and B-vitamin status. Importantly, the sampling procedure should be standardized to reduce analytical variation due to exercise, which is often neglected in clinical practice (Elli et al., 2024; Sanchis-Gomar and Lippi, 2014). At the same time, because patients with CD are at increased risk of osteoporosis due to nutrient loss, regular bone density assessments are recommended (Lucendo and García-Manzanares, 2013).

It is crucial in this population to monitor indicators of nutritional deficiencies or complications of CD. Although a growing body of research exists on CD and its management, extensive guidance for athletes in this circumstance does not exist. There is a lack of discussion in the literature of the unique nutritional requirements and challenges faced by athletes with CD. Athletes, however, are not a homogeneous group: nutritional risk, resource availability, and reporting behaviour differ by sport type (endurance vs. power vs. team disciplines), competitive level (recreational, collegiate/amateur, professional), age category, and sex, and these differences are highlighted throughout the review where the underlying literature allows. This review is designed to give an informed, practice-oriented overview and not definitive clinical advice, due to the limited amount of evidence-based research which is available in relation to athletes. To the best of our knowledge, few existing reviews integrate nutritional management, nutritional supplementation, and functional performance monitoring within a single, athlete-centred review of celiac disease, a gap this review aims to address.

## 2 Methodology

The narrative review was performed according to the Scale for the Assessment of Narrative Review Articles (SANRA), a validated instrument to ensure methodological transparency, clear purpose, and quality of the narrative review (Baethge et al., 2019).

### 2.1 Search strategy

The structured literature search was performed using four main databases: PubMed/MEDLINE, Cochrane Database, BioMed Central, and the BMJ journal collection, from database inception through October 2025. Medical Subject Headings (MeSH) and free-text keywords were used in the search: "celiac disease" OR "coeliac disease" AND "athletes" OR "sports nutrition" OR "athletic performance", "gluten-free diet" AND "micronutrient deficiency" OR "nutritional monitoring", and "celiac disease" AND "monitoring strategies" OR "supplementation" OR "performance". Search terms were combined using Boolean operators (AND, OR) in a systematic way. Additional studies not identified by electronic searches were identified through retrieval of articles from reference lists of retrieved articles and relevant reviews and then screened manually.

### 2.2 Inclusion and exclusion criteria

Inclusion criteria included: (1) peer-reviewed articles published in English; (2) studies that included nutritional considerations, clinical management, or athletic performance in people with CD; (3) studies that included systematic reviews, meta-analyses, randomized controlled trials, observational studies, clinical guidelines, and studies that examined adherence to a gluten-free diet, micronutrient status, supplementation, and performance monitoring for athletic populations. The exclusion criteria included: articles that were not peer-reviewed, editorials and opinion pieces, conference abstracts without a full-text report, single case reports (unless they offered specific and novel information on the nutritional issues of the celiac athlete), and articles not in English and lacking any translation. Where the level of competition (recreational, collegiate/amateur, professional) and age category (adult, paediatric/adolescent) were specified where possible, they are described in the relevant sections; the practical recommendations made in Table 1 should be interpreted according to each source literature as being most applicable to adult, competitive (collegiate-to-professional) athletes, as this is the population from which the majority of evidence was derived.

**Table 1:** Narrative synthesis of nutritional considerations and practical, non-guideline suggestions for athletes with CD.

Category	Nutrient	Challenges/deficiencies in CD athletes	Recommended intake & strategies	Dietary sources & supplements	Key References
Macronutrients	Protein	Impaired intestinal absorption; GI protein losses due to villous atrophy; elevated whole-body protein catabolism; depleted BCAAs and tryptophan.	1.6–2.2 g/kg/day; toward upper range during periods of active disease or incomplete mucosal recovery; distribute 20–30 g per meal every 3–4 h to optimize MPS	Lean animal proteins (poultry, beef, seafood, eggs); legumes, nuts, seeds; pseudo-cereals (quinoa, amaranth, buckwheat); hydrolyzed or pea protein supplements (lactose-free).	Jäger et al. (2017); Braamskamp et al. (2010)
	Carbohydrates	GFD often contains higher GI carbohydrates; risk of fructose intolerance; GI distress during exercise; rapid blood glucose fluctuations.	3–12 g/kg/day (sport-specific). Pre-exercise: 1–4 g/kg (low-GI). During: 30–90 g/h. Post: 1–1.2 g/kg/h within 4 h.	Low-GI GF options (quinoa, rice, potatoes, GF oats); glucose-fructose (2:1) or maltodextrin formulations; hydrogels and HBCD for GI tolerance; GFCO-certified products.	Amawi et al. (2024); Naderi et al. (2023); Cao et al. (2025)
	Fat	GFD is often higher in total fat and SFA; compromised fat-soluble vitamin absorption; limited ALA-to-EPA/DHA conversion (≈0.2%/0.05%).	20–35% of total daily energy; prioritize anti-inflammatory unsaturated fats; 1–3 g/day combined EPA + DHA.	Fatty fish (salmon, mackerel), avocado, nuts, seeds, olive oil; certified GF fish oil or algal-based omega-3 supplements.	Tomczyk et al. (2023); Cannataro et al. (2023)
Micronutrients	Iron	Prevalence up to 82% in CD; impaired absorption; hepcidin elevation (30–50% absorption reduction) post-exercise; only ∼50% restore levels within 1 year on GFD.	1.3–1.7× RDA (∼11–14 mg/day men, 23–30 mg/day women); time intake to early morning; avoid 3–6 h post-exercise window; ferritin ≥50 ng/mL before altitude.	Lean red meats, dark leafy greens, legumes, quinoa, amaranth; pair with vitamin C; sucrosomial iron preferred for supplementation (100–200 mg/day under supervision).	Solberg and Reikvam, 2023; Nolte et al. (2024)
	Vitamin D	Prevalence 8–88% in CD; impaired intestinal absorption; 30% remain deficient after 2 years on GFD; 23.3-fold higher stress fracture risk at low levels.	2000–6000 IU/day; target serum 25(OH)D 30–50 ng/mL (75–125 nmol/L); severe deficiency: 50,000 IU/week for 4–8 weeks.	Fatty fish, fortified dairy, egg yolks; combine supplementation with calcium, magnesium, and vitamin K2; personalize based on VDBP genotype.	de La Puente Yagüe et al., 2020; Lebovits and Lee, 2023
	Magnesium	Impaired absorption; low Mg content in GFD; sweat/urinary losses during exercise; transient hypomagnesemia; serum Mg poorly reflects body stores.	Increase 10–20% above RDA (∼500 mg/day); take ∼2 h prior to exercise for peak plasma absorption; use divided doses.	Leafy greens, nuts, cocoa, legumes, quinoa, buckwheat; organic forms (citrate, glycinate) preferred over oxide (4% bioavailability).	Tarsitano et al. (2024)
	Calcium	41% deficient at diagnosis; >50% inadequate on GFD; lack of Ca fortification in GF products; increased risk of osteoporosis.	1500 mg/day; distribute intake throughout day (max 500 mg at once); combine with vitamin D for enhanced absorption.	Dairy, GF fortified cereals, nuts/seeds (almonds, sesame, chia), dark leafy greens (kale, watercress), fish (sardines, salmon); gluconate, citrate, or carbonate forms.	Rondanelli et al. (2019); Grabia et al. (2024)
	Zinc	48.3% deficient in CD; low Zn in GFD; training-induced sweat losses; lower circulating levels in athletes despite higher dietary intake.	25–40 mg/day until normalization; upper limit 40 mg/day elemental zinc; re-evaluate after 3 months; avoid >60 mg/day (copper interference).	Lean meats, poultry, fish, shellfish (oysters), eggs, dairy, legumes, nuts, seeds; soaking/fermentation improves bioavailability.	Rondanelli et al. (2019); Devarshi et al. (2024)
	B Vitamins	Folate deficiency 10–85%; B12 deficiency 5–40% at diagnosis; low B-vitamin content in GF products; risk of hyperhomocysteinemia.	B12: 500–1000 μg/day (oral/sublingual); 1000 μg IM for severe cases. Folate: 1 mg/day × 3 months, then 400–800 μg/day maintenance.	Lean meats, fish, eggs, dairy, legumes, nuts, seeds, GF pseudo-cereals, fortified GF products; daily GF multivitamin/mineral (∼100% DRIs).	Heffernan et al. (2019); Rondanelli et al. (2019)
Supplements	Probiotics	Residual dysbiosis on GFD (diminished Bifidobacterium); persistent GI symptoms (33% self-initiate use); impaired intestinal barrier integrity.	Multi-strain formulations; minimum 6-week intervention duration; adjunctive to GFD (not a substitute); 29% greater GI symptom reduction vs. placebo.	*Bifidobacterium breve*, *B. animalis* subsp. lactis, *Lactobacillus casei*, *L. plantarum*; multi-strain over single-strain products.	Laterza et al. (2025); Seiler et al. (2020)
	Glutamine	∼7% lower plasma glutamine in active CD; compromised mucosal integrity; increased intestinal permeability; impaired tight junction proteins.	0.3–0.5 g/kg/day; practical upper limit of approximately 30 g/day in clinical contexts; supports intestinal barrier function; not a substitute for GFD	Alanyl-Glutamine (Ala-Gln) dipeptide form preferred for superior stability and bioavailability; upregulates claudin and occludin expression.	Wang et al. (2025); Sevinc et al. (2016)

Abbreviations: CD, celiac disease; GFD, gluten-free diet; GI, gastrointestinal; GF, gluten-free; MPS, muscle protein synthesis; BCAAs, branched-chain amino acids; SFA, saturated fatty acids; EPA, eicosapentaenoic acid; DHA, docosahexaenoic acid; ALA, alpha-linolenic acid; RDA, recommended dietary allowance; HBCD, highly branched cyclic dextrin; GFCO, gluten-free certification organization; VDBP, vitamin D-binding protein; DRIs, dietary reference intakes.

### 2.3 Study selection and data extraction

Records retrieved were independently screened by two reviewers. Titles and abstracts were screened for relevance, then the full text of potentially relevant articles was critically evaluated. Disagreements were settled by discussion and consensus. The evidence was hierarchically classified as highest evidence being systematic reviews, randomized controlled trials, and clinical guidelines; the second-highest evidence being well-designed observational studies; and the lowest evidence being all other evidence (Munn et al., 2018).

The results of data extraction were presented narratively, and themes were arranged under the following headings: nutritional deficiency related to celiac disease, considerations for macronutrients and micronutrients, strategies used for supplementation, challenges of following a gluten-free diet, psychological and socio-economic implications, and strategies for monitoring performance. This review was conducted as a structured narrative review with adherence to the SANRA principles for the improvement of transparency and methodological clarity. Where feasible, descriptive elements commonly used in systematic reporting (e.g., number of studies identified, study design, population characteristics) were reported to enhance transparency and reproducibility; however, this review was not designed as a systematic review and did not include formal study appraisal, risk-of-bias assessment, or meta-analysis. Conclusions from this review should therefore be seen as narrative, integrative, and hypothesis-generating and are not to be interpreted as graded recommendations (Page et al., 2021). The narrative nature of this review required no quantitative synthesis, risk-of-bias assessment or formal evidence tabulation. One of the drawbacks in the literature is the lack of studies that are specific to athletes with actual celiac disease, as determined by biopsy. Several practical considerations discussed in this review are extrapolations from studies of the general celiac population, of athletic populations without celiac disease, and from mechanistic studies or selected case-based reports. The limitation should be taken into account when interpreting the recommendations for the athlete. Reflecting this novelty, the review does not just focus on the conventional nutritional synthesis but also brings together functional performance-based monitoring protocols, such as the timing of biomarkers, body composition measurements, neuromuscular testing, and patient-reported outcomes in a single integrated framework along with nutritional and supplementation recommendations. The integrative, athlete-centred perspective, rather than new primary data, is the principal contribution of this review.

## 3 Prevalence of CD among athletes

Beyond athletes with a confirmed diagnosis, a considerably larger group experiences unconfirmed gluten-related symptoms or follow a gluten-free diet without biopsy or serological confirmation, spanning a spectrum from potential/latent CD to non-celiac gluten/wheat sensitivity (NCGWS); although these individuals fall outside the diagnostic definition of CD used in this review, their overlapping symptomatology and dietary behaviour are relevant to how prevalence is estimated and should be considered when interpreting self-report-based figures (Abdi et al., 2023).

Against this backdrop, Leone et al. (2020) provided an athlete-focused snapshot by surveying 141 National Collegiate Athletic Association (NCAA) athletes using two complementary approaches: self-report of a physician diagnosis and the Celiac Symptom Index (CSI); α = 0.93 (Leone et al., 2020). In this cohort, 4 athletes (≈2.8%) reported a prior CD diagnosis and 16 (≈11.3%) met the study threshold for a high degree of CD-consistent symptoms (CSI ≥ 45) (Leone et al., 2020). Relative to a prespecified general-population benchmark of ∼0.8% (1:125; median age 45 years), the odds of reporting a diagnosis and of high-symptom status were OR 3.85 (95% CI 0.42–34.89) and OR 18.36 (95% CI 2.40–140.48), with significant chi-square tests (Leone et al., 2020). These findings provide useful prevalence indicators in the athlete literature. Interpretation, however, should be cautious: outcomes were self-reported (no serology/biopsy), and the study relied on a limited U.S. (Leone et al., 2020). Convenience cohort recruited in 2014–2015 (<1% of NCAA athletes) with regional (Midwest 48%, Northeast 24%) and demographic skew (70% White), and comparisons were made to the general population rather than age-matched collegiate non-athletes (Leone et al., 2020).

Consequently, while the data suggest that athletes may report CD-consistent symptoms more often than population expectations, these signals should be viewed as provisional rather than definitive prevalence. The true prevalence of CD among athletes, particularly outside North America and Western Europe, remains unknown, as no large-scale, multicenter epidemiological studies employing standardized serological screening and biopsy-confirmed histological diagnosis have been conducted in athletic cohorts. Moreover, the unique physiological context of intense training, including exercise-induced increases in intestinal permeability and systemic immune modulation, may differentially influence both CD expression and diagnostic sensitivity, warranting athlete-specific epidemiological frameworks rather than simple extrapolation from general population estimates (Costa et al., 2017; Leone et al., 2020). Definitive estimates will therefore require adequately powered, multicenter studies using standardized serologic screening and biopsy confirmation, with age- and gender-matched non-athlete comparators to yield precise, generalizable prevalence estimates.

## 4 Genetic, environmental, and physiological factors in CD among athletes

### 4.1 Genetic susceptibility (HLA and Non-HLA loci)

New studies have started to address the complexity of interactions between genetic susceptibility and environmental factors in the development of CD (Pes et al., 2019). Although it has been discussed a lot in gastroenterology and the nutritional sciences, there is a significant lack of literature on how these factors in particular impact athletes. With the specific genetic susceptibility and the physiological strain imposed by sports activity, this field is still under-explored in the field of sports medicine literature. Intense physical stress on athletes may worsen or provoke latent genetic vulnerabilities to CD (Leone et al., 2020). Recurrent gastrointestinal ischemia because of endurance exercise is another type of stress that has been linked to an increase in intestinal permeability, with a similar celiac-type pathophysiology at the beginning (Costa et al., 2017). Athletes represent a distinct population, characterized by specific dietary demands, rigorous training protocols, and unique psychological pressures that together warrant dedicated scientific investigation separate from the general population (Leone et al., 2020).

The high prevalence of CD among athletes is also a genetic phenomenon; studies show that the presence of HLA-DQ2 and HLA-DQ8 alleles is associated with the risk of developing CD (Lázár-Molnár and Snyder, 2018). CD patients are homozygous for HLA-DQ2 (also known as HLA-DQ2.5) in about 90-95%, and heterozygous for HLA-DQ2.5/DQ2.2 in about 5-10%; a very small proportion of patients may be homozygous for HLA-DQ2.2 alone, which confers a smaller but still significant risk (Martina et al., 2018). These genetic markers, however, are not necessarily predictive of CD, as there are environmental factors that are also important (Pes et al., 2019). These HLA types are estimated to be present in about 30-40% of the general population, but only 1-3% will develop CD, meaning that there is a complex relationship between these HLA types and the environment (Kuja-Halkola et al., 2016; Sallese et al., 2020). Although the HLA genes have been identified as playing a role in the development of CD, more than 40 non-HLA genetic loci have been linked to increased risk for CD (Sharma et al., 2016). These are sites of several biological functions such as immune regulation, barrier function of intestines, and inflammatory responses. In addition to HLA-DQ2 and HLA-DQ8, other genes may modify CD, such as Interleukin-2 (IL-2)-IL21, Regulator of G-protein Signaling-1 (RGS1), and Cytotoxic T-Lymphocyte Associated Antigen 4 (CTLA4) (Salazar et al., 2017). These genes do not directly cause CD but contribute to the pathogenesis of CD by promoting immune dysregulation or increasing intestinal permeability.

However, environmental factors also contribute to the development of CD and can even interact with genetic susceptibility to affect the onset and course of CD (Pes et al., 2019; Kuja-Halkola et al., 2016). The overall heritability of CD is high (75%), and non-HLA genetic factors are responsible for 68% of the disease risk, while the environment plays a significant role (32%). This environmental factor, shared and non-shared (17% and 15%, respectively), highlights the need to further explore factors other than genetics that can affect the development of CD (Kuja-Halkola et al., 2016). Given the complexity of the genetics and environment that play a role in the risk of CD, a wide range of environmental factors (Morrell and Melby, 2017) should be investigated, especially in relation to exercise and diet in athletes (Leone et al., 2020).

### 4.2 Environmental and exercise-related factors

Recent years have seen dietary factors and other environmental exposures, including increased focus on the role of gluten, identified as significant contributors to CD risk in athletes, and the traditional gluten-only approach has now become apparent as it offers a limited view. The diet of athletes is usually high in carbohydrates and may contain a high content of gluten-containing foods, and this has long been thought to be a risk factor for the manifestation of CD in genetically susceptible individuals (Olshan and Leonard, 2023). But more recent studies call this into question, as there are more complex factors (Leonard and Fasano, 2019; Morrell and Melby, 2017). There is no consistent correlation between countries with high wheat consumption and high CD prevalence rates and those with low wheat consumption and low CD prevalence rates; for example, Italy and Greece versus Finland (Lionetti and Catassi, 2014). The paradox of this suggests that variations in CD prevalence cannot be explained solely by gluten consumption (Morrell and Melby, 2017). There are a number of other factors that have become known as potential risk factors for CD, which we should recognize when talking about athletic health. The influence of the environment is important, but genetic factors are very important baseline factors. These encompass exposure to infectious agents (Serena et al., 2019), antibiotic use (Leonard et al., 2015), vitamin D status (Infantino et al., 2022), and regional variations as well as the gut microbiome (Leonard et al., 2015). The second is especially interesting because it can be affected by several environmental factors, which can either directly or indirectly affect susceptibility to CD, including cesarean birth (Lupu et al., 2023), formula feeding (Lupu et al., 2023), presence of siblings, pet exposure, and epigenetic pressure (Leonard and Fasano, 2019).

In sports medicine, the physiological requirements of exercise and competition are of a particular nature, and they are interwoven with genetic factors in specific patterns (Leone et al., 2020). These interactions are even more critical in the context of CD and provide new research and understanding opportunities. Strenuous exercise, an integral part of athletes' lifestyle, has been found to have some effects on gastrointestinal functions relevant to CD in recent studies. In a study of runners, for example, the level of serum zonulin was significantly higher following exercise than at rest (p < 0.01) (Sadowska-Krępa et al., 2021). The fact that this finding is important is that zonulin is thought to be a marker of increased intestinal permeability, which plays a significant role in the pathogenesis of CD (Rabiee et al., 2024). Additionally, high-intensity training has been correlated with an increase in expression of the tight junction protein claudin-2 that paradoxically increases the permeability of the intestinal barrier when its expression is elevated, in mice undergoing exhaustive exercise training (Freitas et al., 2020). Importantly, claudin-2 upregulation has been identified to mark active mucosal inflammation in human CD independent of exercise (Villanacci et al., 2024), which could imply that the mechanism underlying exercise-induced gut stress may converge with that of CD in genetically susceptible athletes. The alterations of the gut barrier function associated with claudin-2 expression induced by exercise could raise the likelihood of developing CD in genetically susceptible athletes (Villanacci et al., 2024; Luettig et al., 2015). However, it's important to note that these findings are based on animal studies and may not be directly translated to humans. They nevertheless offer significant information that should be further studied in humans. In the midst of these findings, a new phenomenon is being revealed, which is called Exercise-Induced Gastrointestinal Syndrome (Ex-GIS). This syndrome includes several digestive disorders that may happen during or following endurance and heavy exercise (Ribichini et al., 2023). The symptoms of this syndrome could resemble or intensify the symptoms of CD, making diagnosis and management more challenging in the athlete (Costa et al., 2017). Although these findings indicate potential mechanisms whereby physical activity might impact CD risk or presentation, the direct connection between physical activity and CD development or progression continues to be a research area. The interaction between the genetic, environmental, and physiological stressors of sport participation highlights the need for continued research to elucidate the implications for CD in athletic populations (Leone et al., 2020).

### 4.3 Epigenetic mechanisms

Furthermore, the involvement of epigenetics in the pathobiology of CD is a new research field which may generate more insights into predisposition to CD and disease pathogenesis (Gnodi et al., 2022). Environmental factors may regulate gene expression associated with CD susceptibility as a result of epigenetic modifications (Ghosh et al., 2022). Such changes also play a role in the complex relationship between genes and environment in the development of CD (Cielo et al., 2019). Several genes and genomic regions were identified in which the CD patients' DNA methylation pattern has been shown to vary from that of healthy controls. Differential methylation has been reported for genes related to pathways that regulate inflammation in CD (Nuclear Factor-κB (NF-κB) pathway), intestinal barrier function (SH2B Adaptor Protein 3 (SH2B3) pathway), and immune function (IL21 pathway) (Gnodi et al., 2022; Ghosh et al., 2022). Furthermore, cell-specific methylation modifications have been detected in both epithelial and immune cells of CD patients (Gnodi et al., 2022; Cielo et al., 2019). The influence of, for example, athletes' diet and exercise on the epigenetic signatures of CD will need to be explored, but these epigenetic signatures could be used as patient stratification or disease risk markers, especially in high-risk populations like athletes (Tan et al., 2021).

This increase in CD in athletic populations might be due to increased vigilance and diagnostic testing, but not because athletes have an inherent risk of developing CD (Leone et al., 2020). Although there is a genetic predisposition, there is no definite correlation between the severity of physical exercise and the worsening of CD, while the triggering factor is gluten. Taking a detailed account of these factors is essential to designing successful interventions to maintain health and maximize the functioning of athletes with CD. The use of genetic screening as a component of a comprehensive health examination of an athlete for the presence of a CD genetic predisposition, or other autoimmune disease, may be considered seriously, especially in athletes whose family history includes CD or other autoimmune disease. Genetic testing does not always help to predict the likelihood of CD, but it can help with personalized monitoring plans and early intervention (Rubio-Tapia et al., 2023).

## 5 The multifaceted challenges and effects of CD on athletic performance

### 5.1 Psychological and mental health challenges

The intricate connection between CD and athletic performance presents a complex psychological picture, especially concerning increased risks of anxiety and depression. Recent studies have clearly shown that CD has a profound effect on mental health, not just its gastrointestinal symptoms, particularly in the stress field of competitive sports. There are few studies specific to athletes with CD; however, there is some research on the general population of children with CD that can be tentatively extrapolated to the athletic setting (Lee et al., 2021; Clappison et al., 2020). A meta-analysis by Clappison et al. (2020) reported that individuals with CD face significantly higher odds of experiencing anxiety (OR: 6.03, 95% CI: 2.22-16.35, P < 0.0001) and depression (OR: 2.17, 95% CI: 2.17-11.15, P < 0.0001) (Clappison et al., 2020). The results highlight the significant psychological impact of CD, which may be exacerbated by the competitive atmosphere of sports (Fig. 2). Consistent with this, athlete data show that higher CSI scores are moderately to strongly linked to poorer Health-Related Quality of Life (HRQOL) across the physical (r = −0.49), psychological (r = −0.53), social (r = −0.38), and environmental (r = −0.36) domains, and to greater depression (Beck Depression Inventory; r = 0.62) and higher perceived stress (PSS-14; r = 0.53). Together, these associations highlight a meaningful psychosocial load in athletes with more CD-consistent symptoms. (Leone et al., 2020). The psychological impact of CD appears to be enduring and may originate in early life stages. A population-based cohort study revealed that childhood CD was associated with a 19% increase in the risk for any psychiatric disorder (95% CI, 1.14–1.23), including elevated risks for mood and anxiety disorders (Lebwohl et al., 2021). Importantly, this increased risk for psychiatric disorders continued into adulthood (hazard ratio, 1.11; 95% CI, 1.04-1.17) (Lebwohl et al., 2021).

**Fig. 2:**
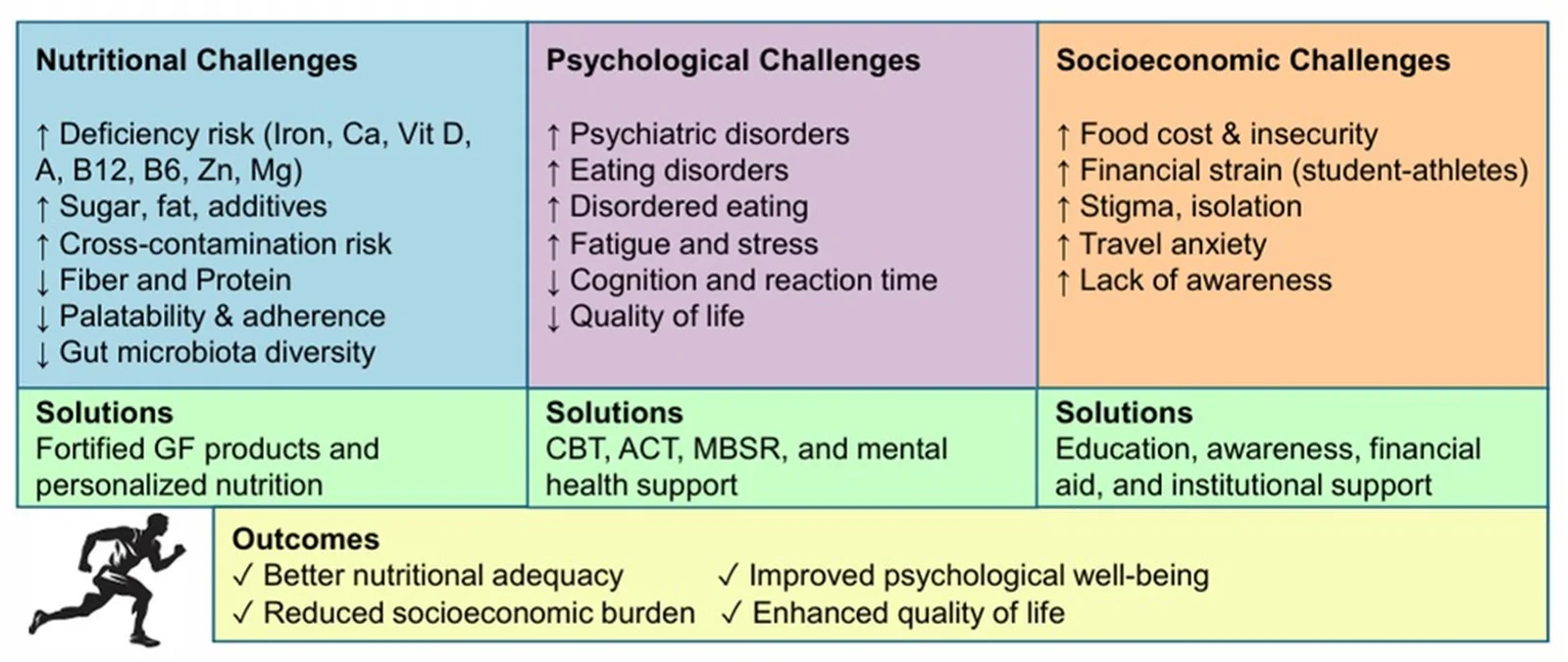
Nutritional, psychological, and socioeconomic challenges of CD athletes.

The mechanisms underlying the link between CD and mental health issues are multifaceted and not yet fully understood. Theories encompass a range of factors, including nutrient deficiencies (such as vitamin B12, B6, and folate) due to malabsorption, autoimmune reactions affecting brain function, alterations in gut microbiota influencing the gut-brain axis, cerebral hypoperfusion, and psychosocial factors related to managing a chronic condition (Cossu et al., 2017; Campagna et al., 2017). In the context of sports, these physiological processes can work together with the physical and psychological demands of the activity to further hinder recovery processes and performance, and raise the likelihood of sport-related injuries (Rogers et al., 2024). On top of all these psychological issues, the food issue with CD is another major clinical/psychological concern. A systematic review and meta-analysis of eating disorders in CD cases were conducted and showed a significant rate of eating disorders (pooled prevalence 8.88%, 95% CI: 6.4–11.7) (Nikniaz et al., 2021). Importantly, these conditions are bidirectional in that the risk of developing anorexia nervosa was significantly higher in patients with CD than in healthy controls (RR: 1.48, 95% CI: 1.32-1.64) (Nikniaz et al., 2021). This increased risk is attributed to several factors, including the challenging nature of a strict GFD, increased food insecurity, and psychological distress; studies have demonstrated that CD patients who developed an eating disorder displayed binge/restrict cycles and heightened body image concerns (Satherley et al., 2017; Fraser et al., 2022). Although eating disorders and disordered eating are similar, they are not the same; eating disorders are clinically diagnosed conditions outlined in the DSM-5, such as eating disorders (anorexia nervosa and bulimia nervosa, and disordered eating is a broader range of unhealthy eating habits that do not meet full diagnostic criteria but may still impact health and performance. Considering the high prevalence of athletes with disordered eating and the high prevalence of athletes who participate in anaerobic sports with disordered eating, the combination of athletes and CD could be a compounded risk profile that needs specific attention and research.

In addition to mood disorders and eating issues, cognitive function and mental preparedness are important considerations in the CD-athletic performance relationship. The phenomenon, often referred to as 'brain fog,' involves measurable deficits in sustained attention, information processing speed, and executive functioning following validated neuropsychological assessments and confirmed by neuroimaging showing white matter microstructural change, with implications for athletic performance including sports requiring rapid tactical processing, decision-making under fatigue, and reaction-time precision (Croall et al., 2020; Yelland, 2017). More recently, Croall et al. (2020) demonstrated significant deficits in reaction time alongside microstructural changes in white matter integrity in individuals with CD compared to healthy controls, providing neuroimaging evidence that corroborates earlier reports of gluten-related cognitive impairment (Yelland, 2017). The causes of these cognitive changes are complex and may include nutrient deficiencies (such as iron, B12, folate, and vitamin D) and systemic inflammation. A rise in pro-inflammatory cytokines has been linked to changes in behavior, mood, and cognition, which may result in decreased velocity of neuronal transmission and slowdown of information processing speed (Makhlouf et al., 2018; Ravikumar et al., 2022).

The most important treatment for CD is a GFD, which has been shown to have beneficial effects on psychological well-being (Moawad et al., 2024). In a meta-analysis that analyzed the effects of a GFD on anxiety, results showed that anxiety scores significantly decreased after 1 year of following a GFD. State anxiety, assessed by the State–Trait Anxiety Inventory, Form Y-1 (STAI-Y1), decreased significantly (MD: 3.48, 95% CI: [0.26, 6.71]), as did trait anxiety, assessed by the State–Trait Anxiety Inventory, Form Y-2 (STAI-Y2) (MD: 3.45, 95% CI: [1.39, 5.52]) (Moawad et al., 2024). But keeping to a strict GFD presents a number of challenges, especially for athletes who can find themselves in a situation of dietary complexity when traveling, competing, and eating out with teammates (Wieser et al., 2021a, Wieser et al., 2021b). This extra control of what to eat and drink may increase stress and anxiety. Despite being necessary for managing CD, however, extreme vigilance in maintaining a GFD can actually worsen symptoms of CD, including anxiety and fatigue, and lower QOL (Wolf et al., 2018). Although no specific psychological interventions have been studied in athletes with CD, there are some insights to be gained from the behavioral interventions that have been studied for chronic digestive conditions. Adapted psychological interventions, such as Cognitive Behavioral Therapy (CBT), Acceptance and Commitment Therapy (ACT), and Mindfulness-Based Stress Reduction (MBSR) are effective in some cases in managing food intolerance-related issues (Palsson and Ballou, 2020; Wynne et al., 2019; Aucoin et al., 2014; Cherpak, 2019). There are positive outcomes of these interventions, such as decreased symptom-specific anxiety, hypervigilance, and disordered eating behaviours, and enhanced HRQOL in a range of gastrointestinal conditions (Coburn et al., 2022). CBT techniques, focused on reframing counterproductive thoughts about dietary restrictions and reducing catastrophizing, combined with ACT and MBSR approaches that emphasize present-moment awareness and values-based activities, may be particularly relevant for athletes managing CD (Nikoo Goftar et al., 2019; Sabzevari et al., 2023; Petterson and Olson, 2017).

### 5.2 Nutritional challenges

Adopting GFD as a routine dietary habit effectively manages CD symptoms and complications (Al-Toma et al., 2019). However, it poses significant challenges, particularly for athletes with heightened nutritional demands (Fig. 2). Typically, a GFD excludes gluten-containing grains such as barley, rye, and wheat, resulting in lower dietary fiber intake (Abdi et al., 2023). Consequently, excluding these grains may lead to micronutrient deficiencies compared to gluten-containing diets (Miranda et al., 2014; Calvo-Lerma et al., 2019). The poor nutritional quality of Gluten-Free Products (GFP), notably the lack of iron and calcium fortification, further exacerbates these deficiencies (Herrera-Quintana et al., 2025). Research has shown that individuals following a GFD are at risk of not getting enough iron, calcium, vitamin D, vitamin A, B12, B6, zinc, and magnesium (Herrera-Quintana et al., 2025). A few deficiencies can be reversed by a GFD, but some, like vitamin A, vitamin D, iron, copper and zinc, may only be partially reversible (Herrera-Quintana et al., 2025). Although there is good clinical evidence of its efficacy, strict adherence to a GFD is highly variable, ranging from 45% to 90%, accounting for methodological differences between studies (dietary recall, dietitian-confirmed or gluten immunogenic peptide biomarker testing), age at diagnosis, and cultural background (Muhammad et al., 2019; Wieser et al., 2021a, Wieser et al., 2021b). The range highlights the limited value of athletes' self-report for monitoring, especially when traveling in teams, competing in restaurants, and sharing kitchens. Other factors such as dietary knowledge, psychological factors, sociocultural background, parental education, occupation, age, gender, and age at diagnosis all play a key role in dietary compliance (Muhammad et al., 2019; Simón et al., 2023).

One of the most important barriers is the lack of availability and access to GFP, which further hinders adherence, particularly in countries such as Brazil (Araújo and Araújo, 2011), Morocco (Guennouni et al., 2022), Saudi Arabia (Al-Sunaid et al., 2021), and Mexico (Arias-Gastelum et al., 2018). Recently, awareness and product availability have improved, but many people do not have access to these specialized products (Demirkesen and Ozkaya, 2022). Frequent training intensity and, of course, the demands of the competitions make these nutritional problems even greater for athletes (Sandvik et al., 2020). Frustration and anxiety over limited accessibility and provision of GF meals in athletic departments and dining services were also reported by collegiate athletes, adding stress and thereby hampering their ability to concentrate on athletic performance (Sandvik et al., 2020). In some instances, the athlete has openly stated that inadequate nutrition before exercise had a direct negative effect on his/her performance (Sandvik et al., 2020). In addition, the general public marketed GFP often lacks the nutritional density of proteins, vitamins and minerals that are desirable by athletes, and it contains more fat, sugar, salt and additives (Melini and Melini, 2019; Staudacher and Gibson, 2015). Furthermore, the decreased palatability of GF foods causes a reduction in dietary enjoyment and long-term adherence, especially in people with CD (Roma et al., 2010; White et al., 2016). Foods made without gluten are different in texture, flavor, and appearance, and frequently can be dry, coarse, and less appealing (Staudacher and Gibson, 2015). In a particular case of a cyclist who developed CD during an ultra-endurance event, these specific dietary difficulties were emphasized, including the difficulty of eating GF foods because they were dry and crumbly, and the difficulty of eating these foods in harsh weather. Furthermore, although energy-dense, GF foods were less satiated because of their low protein content, which might lead to hunger, discomfort, and early-onset fatigue and negatively influence athlete performance (Black et al., 2012). To make up for this, sugar, fat, or additives are typically added to GF foods, creating additional nutrition concerns (Melini and Melini, 2019; Staudacher and Gibson, 2015). Thus, current food science studies are dedicated to improving the nutritional profile, flavor, texture, and appearance of GF food products for improved dietary compliance (Hosseini et al., 2018).

However, there is also a dietary risk due to gluten cross-contamination that is a major challenge for athletes with CD (Wieser et al., 2021a, Wieser et al., 2021b). There have been numerous studies reporting high levels of, and exceeding, the safe gluten level (20 ppm) in meals marketed as GF, especially in shared kitchens, restaurants, and catering service environments (Wieser et al., 2021a, Wieser et al., 2021b). For susceptible people, even small amounts of gluten can cause changes in the intestinal lining. There is no clear consensus on a universally safe daily amount of gluten; recent guidelines suggest that it should not exceed 20 ppm per day to reduce risk (Cohen et al., 2019). Total daily gluten consumption that seems to be safe for most CD patients is < 50 mg of gluten; nevertheless, some CD patients need as little as 10 mg of daily gluten to promote development of intestinal mucosal abnormalities (Cohen et al., 2019). The true 'no-effect' threshold is not the <20 ppm that has been used for GF labelling, as sensitive individuals, such as those with refractory CD and/or incomplete mucosal healing, are at risk of developing histological injury at very low exposure levels. In athletes with a compromised intestinal barrier from ischemic exercise stress and increased permeability, the safe threshold for exposure may be even more restricted, and therefore prudent caution should be taken in choosing what foods to eat during a competition or trip to the races (Lerner et al., 2019; Cohen et al., 2019).

Though these are complex issues, a systematic review of 18 published studies (Burger et al., 2017) found that a very high proportion of people with CD have greater HRQOL when following a strict GFD. Furthermore, a recent case study of a high-level breaststroke swimmer with CD showed that personalized nutritional interventions, specifically the optimization of nutrient timing around training, benefited both the clinical parameters and quality of life, as well as the sporting performance of the athlete for up to 12 months (Cannataro et al., 2023). Other case studies of a female collegiate tennis player also highlight the need for a team-based approach to dietary management to manage symptoms and preserve an athlete's performance and QOL with CD (Leone et al., 2005). However, existing studies focusing on nutritional problems do not specifically focus on athletes. Thus, further specific studies are needed to fully understand the nutritional requirements and issues associated with athletes with a diagnosis of CD.

### 5.3 Socioeconomic Challenges

In addition to individual costs, factors such as the socioeconomic status of the athlete and the economic and policy environment of his or her country of training and competition influence adherence to a GFD for athletes with CD and thus the practical availability and accessibility of gluten-free products and specialist CD dietetic care and diagnostic testing. In the United Kingdom (UK) GFP has consistently been reported to be significantly more expensive than conventional, with prices in the UK reported to be 76–518% higher, in Canada ∼180% higher, and in the US ∼183–240% higher (Demirkesen and Ozkaya, 2022). In the Middle East, the price of GFP was significantly higher than that of conventional foods; in the UAE, the price difference was 259% on average, with a difference of up to 1100% for some products like bread mixes (Abdulla and Garemo, 2018). While in the Kingdom of Saudi Arabia (KSA), prices are often reported to be roughly double those of regular foods (Sarkhy et al., 2016; Qashqari et al., 2024). The budget concern is particularly pressing for young competitive athletes, who have a fluctuating financial situation and at the same time a high demand for nutrition (Lambert and Ficken, 2016). Beyond the cost of the diet, the costs of basic needs like uniforms, equipment, league fees, travel, and footwear also place a significant burden on the athlete and their family (Merkel, 2013). These increased costs are a key driver of "gluten-free food insecurity" (Ma et al., 2022), experienced by about one in six people with CD regularly, and which has an impact on dietary adherence. This relationship has been empirically supported; Elsahoryi et al. (2024) found in 1162 adults with CD that food insecurity significantly impairs HRQOL, with GFD adherence mediating this effect—only 8.3% achieved excellent adherence, and 71.9% reported poor HRQOL levels. Moreover, as dietary expenses are not the only costs associated with CD, there are also significant indirect costs incurred with comorbidities and complications associated with CD that lead to greater healthcare utilization (Bozorg et al., 2024). In addition, young people with CD face health care costs that are disproportionately high relative to their peers, which places a disproportionate burden on their finances (Bozorg et al., 2024). In addition, there are regional inequities in access to and institutional support for these challenges (Bozorg et al., 2024). Although some GFP is available in hospital settings, there are still people who are unable to access affordable GFP in KSA (Qashqari et al., 2024). In contrast, there are many financial support mechanisms in North America and Europe, but not as widespread in other parts of the world, including in the Philippines (Bozorg et al., 2024). For example, in the United States and Canada, there is a tax deduction policy; in Italy, a monthly stipend; and in Norway and Sweden, a reimbursement policy (Bozorg et al., 2024). In the UK, the GF staples are supported by prescription (Kurien et al., 2018), but recently the support has been limited, with continuing concerns about their affordability (Burden et al., 2015).

These economic costs coincide with the results of Sandvik et al. (2020), who found that student-athletes specifically reported that there was a substantial financial burden related to purchasing GF foods independently (Sandvik et al., 2020). In the social domain, collegiate athletes with CD report being significantly less socially healthy in their lives (Leone et al., 2020). One of the few qualitative studies focusing on collegiate student-athletes with CD was performed by Sandvik et al. (2020) and revealed significant reports of feelings of isolation because of the inconsistent availability of GF options within the athletic department and campus dining services. This is also compounded by challenging sports schedules and a lack of time for meal preparation (Sandvik et al., 2020). This is consistent with the larger community studies that found about 72.6% of people with CD reported high stress at food-related social events and more than half of the larger community sample said they were an inconvenience to others because of their dietary limitations (Stroebele-Benschop et al., 2025). Dietary needs are often underestimated by peers, which makes dietary needs more likely to lead to embarrassment, stigma, and social exclusion (Stroebele-Benschop et al., 2025). These experiences are consistent with concepts from stigma and social support theories, highlighting the psychological impact of perceived social burdens (Pitt et al., 2024; Olsson et al., 2009). There are also a number of logistical challenges faced by athletes, particularly when traveling with a team. This includes a lack of access to key food storage and preparation resources, inconsistent access to meals served in the GF restaurant system, and extra time needed for careful meal planning (Sandvik et al., 2020). These reported experiences are supported by other community-level data, as about a quarter of people with CD say they don't eat out at all because they're worried about exposure to gluten while traveling, and about a third of meals served in restaurants that are labeled GF actually contain detectable gluten (Leffler et al., 2008; Lerner et al., 2019). More than 3/4 say that they were sad and anxious about not being able to enjoy cuisine and culture (Stroebele-Benschop et al., 2025). The combination of these challenges can lead to a high level of anxiety for athletes, which in turn can harm their concentration and performance (Sandvik et al., 2020).

Lack of CD awareness among coaches, athletic trainers, dining staff, and peers is another major obstacle that may leave athletes with CD to educate others about their dietary requirements, increasing social stress (Sandvik et al., 2020). To assist, the student-athletes propose the inclusion of a full-scale CD education in training and a financial investment to reduce economic and sociopsychological burden (Sandvik et al., 2020). These financial supports might include direct subsidy, reimbursement of meal plans, or food vouchers. Coaches, dietitians, and trainers are all members of the interdisciplinary team and are vital to educating and supporting athletes proactively to reduce the amount of social and logistical stress (Ralphs and Piper, 2016). In addition, dietary adherence and social stress linked to the disease can be further enhanced through increased participation in organized celiac associations and the provision of structured education and social support, which has been the subject of dedicated research (Abu‐Janb and Jaana, 2020). Furthermore, good family involvement is associated with better dietary compliance and emotional health (Ludvigsson et al., 2015). Digital resources and social media can be very helpful; however, misinformation can be shared online and worsen anxiety (McNally et al., 2012). Focusing on the economy, direct subsidy programs and reimbursement for meals, as used in countries such as Italy, Norway, KSA, and Sweden, could improve adherence to a GFD (Bozorg et al., 2024). Application of such support systems on a global level would significantly lower the economic barriers, increase athlete dietary compliance and, in turn, the social and logistics barriers (Fig. 2).

### 5.4 Approaches from other chronic gastrointestinal conditions

Athletes with CD face several similar practical and psychosocial challenges as athletes with other chronic gastrointestinal diseases (e.g., inflammatory bowel disease [IBD] and Crohn's disease), such as iron-deficiency anaemia, dietary restriction due to their disease and increased psychological distress. Structured, condition-specific management approaches developed in IBD may offer transferable models for CD athletes: the European guidelines for IBD recommend systematic monitoring for iron deficiency and anaemia and propose iron-repletion protocols according to disease activity (Dignass et al., 2015), an approach similar to the iron-monitoring strategy described for CD athletes in Section 7. Likewise, psychological training (acceptance and commitment therapy) was found to be effective in lowering psychological stress associated with the disease in people who suffer from IBD (Wynne et al., 2019), indicating that formal psychological support, rather than provision of counselling only at the time of need, may also be effective in reducing anxiety and/or social burden in athletes with CD associated with dietary restriction (Section 5.1). Direct extrapolation should be done with caution, however, since the mechanisms of action of these two diseases, the treatment (pharmacological therapy in IBD, dietary exclusion in CD), and the course of the disease differ.

## 6 Nutritional and supplements consideration for athletes with CD

Nutrition plays a central role in athletic performance, recovery, and the reduction of injury and illness risk (Amawi et al., 2024). Central to sports nutrition is Energy Availability (EA), which refers to the energy remaining for essential physiological processes after accounting for energy expended during exercise (Amawi et al., 2024). Optimal EA management is especially critical for endurance athletes, who typically require 50–80 Kilocalories (kcal) per Kilogram (kg) body weight/day (Kerksick and Kulovitz, 2013), whereas strength and power athletes such as sprinters and weightlifters generally require approximately 44–50 kcal/kg body weight/day due to their high-intensity activities (Kerksick and Kulovitz, 2013). Individual variability influenced by age, sex, training status, and sport-specific demands must also be considered.

Athletes with CD must strictly adhere to a GFD (Al-Toma et al., 2019), which often results in deficiencies in protein, fiber, iron, B vitamins, zinc, vitamin D, magnesium, and calcium (Kreutz et al., 2020; Hart et al., 2011; Shepherd and Gibson, 2013; Vici et al., 2016; Cardo et al., 2021). These deficiencies raise the likelihood of Low Energy Availability (LEA), which can result in Relative Energy Deficiency in Sport (RED-S), including hormonal issues, compromised bone health, compromised immunity, and adverse cardiovascular, psychological, and gastrointestinal effects (Gallant et al., 2024). Around 22–58% of athletes suffer from LEA symptoms, highlighting the importance of nutritional interventions that need to be made proactively (Logue et al., 2020).

Personalized nutrition strategies need to be developed that are based on individual physiological profiles and nutritional needs to effectively respond to these nutritional demands. The concept of personal nutrition is still fairly new, yet it has roots in ancient Medical Dietetics (Mayes and Meloni, 2024). A few recent studies have pointed out the shortcomings of a generic GFD without personalization. In other cases, Vereczkei et al. (2021) reported low levels of fat-free mass and high levels of fat mass (Vereczkei et al., 2021) in CD patients after a non-personalized GFD, impacting their body composition. These negative alterations are especially troublesome for athletes of weight-dependent sports, where careful regulation of body composition is essential to maximize performance. Since there have been no published clinical studies specifically focused on the nutritional aspects of personalized nutrition in athletes, there is the potential to learn from related populations. Women with CD had a randomized 12-wk intervention combining a personalized plan of GFD with supervised RT, with statistically significant positive results in body composition, urogenital symptoms and bone-related indices, and in mood (higher vigor) measures when compared to non-intervention comparators (Martínez-Rodríguez et al., 2022).

The same direction was observed in a randomized trial performed in malnourished seniors, where a diet tailored to individual needs and a structured exercise program (or no exercise) over 12 weeks consistently improved nutritional status, physical function, and quality of life (Arici et al., 2025). Collegiate athletes provide additional qualitative evidence of the benefits of personalized nutrition plans, which include improvements in their athletic performance, recovery, energy availability, overall well-being, and mental clarity and focus (Stavitz and Koc, 2024). Taken together, these results indicate that combining personalized nutrition with specific exercise could provide beneficial effects on health, body composition, and performance. In addition, recent developments in sports nutrition have highlighted the potential significance of personalized approaches in sports nutrition for individuals with CD, where careful monitoring of nutrient status in relation to strict dietary requirements and performance objectives is crucial (Sutehall and Pitsiladis, 2025). Concurrently, significant genetic, physiological, and metabolic variability among individuals presents a problem for the effectiveness of a “one-diet-fits-all” approach (Roman et al., 2024; Guest et al., 2019; Plotnikoff, 2014). Overlooking these differences will only exacerbate the existing nutrition deficiencies and impair athletic function, particularly in susceptible populations, including athletes with CD. Although significant progress has been made, there is an urgent need for well-designed randomized controlled trials in athletes in general and CD athletes in particular to provide guidance and to directly assess nutritional status and performance outcomes to verify the evidence. The combination of individual genetic, physiological and lifestyle aspects into the planning of an athlete's diet can provide a nutritional approach to manage nutritional risks, minimize unwanted side-effects and facilitate athletic performance and quality of life.

### 6.1 Macronutrients

#### 6.1.1 Protein

Protein is important to support muscle repair, growth and recovery, especially when athletes are doing high-intensity exercise as their protein needs are higher compared to those of athletes doing lower intensity exercise (Jäger et al., 2017). Individuals with CD have specific nutritional needs, which include a reduced rate of intestinal protein absorption (Hart et al., 2011), the naturally lower protein content of GFD (Miranda et al., 2014; Calvo-Lerma et al., 2019), and gastrointestinal losses of protein caused by villous atrophy and epithelial damage (Rodrigo, 2006). In spite of these defects, there is a marked increase in mucosal protein synthesis in CD, which probably reflects increased mucosal cell turnover and increased protein breakdown (Nakshabendi et al., 1996). New studies have demonstrated that protein catabolism is significantly higher in active CD, particularly relating to the use of essential amino acids, like leucine and glutamine, which may worsen deficiencies (Messing et al., 1998). Altered plasma amino acids, with depleting tryptophan and branched-chain amino acids (BCAA) are also emerging in metabolomic analyses during active CD and also add to the challenge of muscle protein synthesis and repair (Vacca et al., 2022). In addition, the absorption of amino acids is significantly reduced in CD patients without treatment compared to healthy people, especially L-alanine (p < 0.05) and glycine (p < 0.01) (Yamanaka-Okumura et al., 2023).

The compounding negative effects of chronic mucosal inflammation, villous atrophy-induced malabsorption, and increased whole-body protein catabolism in active or poorly controlled CD may warrant protein intake closer to the high end of the recommended sports nutrition guidelines (1.6–2.2 g/kg/day for all but elite athletes); however, this extrapolation is speculative in the absence of CD-specific athlete interventional data and should be tailored based on individual clinical assessment (Jäger et al., 2017; Braamskamp et al., 2010). There are indications from protein-losing enteropathies as well as from clinical situations involving compromised gut from use for conditions that higher protein intake is warranted in select cases, but these ranges should be used with caution in athletes with CD due to the lack of direct interventional evidence (Braamskamp et al., 2010).

Thus, athletes with CD can benefit from consuming a variety of high-quality, GF protein sources, such as lean animal protein foods (e.g., seafood, poultry, beef, eggs), plant protein foods (e.g., legumes, nuts, seeds), and nutrient-dense pseudo-cereals (e.g., quinoa, amaranth, buckwheat) (Ralphs and Piper, 2016; Eberman and Cleary, 2005; Caeiro et al., 2022). If supplementation is recommended, hydrolyzed or plant proteins like pea protein may be beneficial, especially if gastrointestinal tolerance or absorptive capacity is a concern (Koopman et al., 2009). Furthermore, it is important to note that, in some people, lactose intolerance can also develop in individuals with current CD, so in certain cases, a lactose-free protein product might be more suitable (Ojetti et al., 2005). Distributing protein intake evenly throughout the day approximately 20–30 g per meal every 3–4 h is recommended to optimize amino acid availability, maximize muscle protein synthesis, and mitigate the absorption challenges associated with active CD (Jäger et al., 2017). However, for maximizing the anabolic response, protein distribution through the day is a minor consideration compared with achieving the targeted daily total (Hudson et al., 2020).

#### 6.1.2 Carbohydrates

Carbohydrates play a crucial role in athletic performance, recovery and fatigue resistance (Amawi et al., 2024). Good quality sources of GF foods to choose from include rice, quinoa, potatoes, certified GF oats, fruits and pseudo-cereals, as they are all high nutritional quality foods for athletes with CD (Ralphs and Piper, 2016; Eberman and Cleary, 2005; Caeiro et al., 2022). But glycaemically, GF products, in particular products made with refined rice flour, tapioca starch, and potato starch, are often as high or higher in glycemic index than their gluten counterparts. Based on the evidence presented in the meta-analysis, the mean GI for GF breads are 72.1 compared to 62.6 for a standard wheat bread, which would have implications for athletes with a need for a prolonged supply of energy during long training sessions or during days of the week with multiple events. These fluctuations can lead to overproduction of insulin, reduce fat oxidation, and cause either hypoglycemia or rebound hypoglycemia, which adversely impacts performance (Cao et al., 2025). Hence, controlling blood glucose prior to and during competitions may be a crucial factor to consider (Cao et al., 2025).

The sports nutrition window (3–12 g/kg/day) can be recommended as a daily intake for carbohydrates, depending on the sport, training load, gastrointestinal tolerance, and overall dietary pattern (Amawi et al., 2024). To maximize glycogen storage and reduce gastrointestinal discomfort, pre-exercise carbohydrate intake should be low on the Glycemic Index and low in fibre (preferably GF), such as quinoa, rice, potatoes, GF oats (1-4 g/kg, 1-4 h before) (Naderi et al., 2023). On competition day, athletes are encouraged to steer clear of new or unfamiliar foods that are GF but not certified (Cao et al., 2025; Naderi et al., 2023). For events that last between 1 and 2.5 h, the recommended intake of carbohydrate is 30–60 g/h, and for events longer than 2.5 h, the intake of carbohydrate can be increased up to 90 g/h (Naderi et al., 2023). Carbohydrate blends (usually a 2:1 ratio of carbohydrate sugars—glucose + fructose) may improve the absorption of carbohydrate and minimize gastrointestinal discomfort (Cao et al., 2025; Naderi et al., 2023). Athletes with CD should test these combinations during training carefully, however, because of the possibility of fructose intolerance in those with CD (Ciacci et al., 2006). If this happens, it might be beneficial to use isotonic drinks with maltodextrin (such as corn-derived) that might be absorbed faster and have less gastrointestinal discomfort (Cao et al., 2025; Naderi et al., 2023). In addition, novel carbohydrate formulations like hydrogels and Highly Branched Cyclic Dextrin (HBCD) might help to minimize gastrointestinal symptoms and allow for increased carbohydrate consumption, but should be tested for individual tolerance during training (King et al., 2020; Morenas-Aguilar et al., 2025). There may be a system for gut-training protocols and carbohydrate formulation testing that can be done well before the competition to help determine appropriate carbohydrate sources and amounts.

Carbohydrate replenishment immediately after exercise (1–1.2 g/kg/hour within the first 4 h) with high-glycemic-index (GI) carbohydrate sources that are free of gluten can help replenish glycogen stores quickly (Cao et al., 2025; Naderi et al., 2023). Liquid or gel carbohydrate supplements are preferred during high-intensity and/or long-duration exercise because they are more likely to empty from the stomach quickly and to result in less gastrointestinal discomfort than solid foods (Cao et al., 2025). Athletes with CD should look for GF labeling either from the product or a third party to ensure they are eating a safe food. Finally, environmental factors (such as temperature, humidity, and altitude), gastrointestinal tolerance, and nutritional needs should also be taken into account on an individualized basis to maximize exercise performance and recovery benefits for personalized carbohydrate supplementation (Cao et al., 2025).

#### 6.1.3 Fat

Fat is also a must-have in a healthy diet, as it is a source of energy and helps absorb fat-soluble vitamins (Ravisankar et al., 2015). To achieve sufficient intake of recommended essential fatty acids and vitamins (Amawi et al., 2024), athletes should consider a total fat intake of 20-35% of the recommended daily amount of energy intake. However, GFDs, which are commonly followed by people with CD, tend to have a higher total fat content than normal diets (Melini and Melini, 2019; Staudacher and Gibson, 2015; Marciniak et al., 2021), especially SFA (Cardo et al., 2021; Ballestero-Fernández et al., 2021). Higher intakes of SFA have been linked to a higher risk of cardiovascular and chronic diseases, which may pose extra health risks for people with CD (Sacks et al., 2017). However, if fat intake is too low (less than 20% of energy), this could lead to insufficient absorption of fat-soluble vitamins and adversely affect performance and recovery (Thomas et al., 2016). Nevertheless, planned short-term reductions in fat intake, such as during carbohydrate loading, can be strategically implemented to optimize athletic performance (Wachsmuth et al., 2022). Incorporating anti-inflammatory, unsaturated fat sources such as fatty fish (rich in omega-3), avocado, nuts, seeds, and olive oil can support overall health and recovery in athletes with CD (Tomczyk et al., 2023). However, evidence and recommendations on fat requirements and their effects on performance outcomes in athletes with CD are limited; further CD-specific, athlete-focused studies are needed. Omega-3 supplementation may be of interest in athletes with CD because EPA and DHA contribute to anti-inflammatory and pro-resolving pathways that may support intestinal homeostasis and recovery following gluten-triggered mucosal injury and exercise-induced oxidative stress (Cannataro et al., 2024).

Clinical evidence directly involving athletes with CD supports this rationale, as supplementation with 1.5 g of combined EPA and DHA daily in a high-level swimmer with CD was associated with measurable improvements in inflammatory markers and recovery parameters (Cannataro et al., 2023, 2024). Furthermore, a controlled trial in CD patients showed that 2 g of fish oil daily, providing 420 mg EPA and 230 mg DHA, significantly reduced both C-reactive protein and IL-6 when combined with aerobic exercise, suggesting that the anti-inflammatory benefit is amplified rather than diminished in physically active individuals with CD (Costa and Brito, 2022).

Due to the limited conversion of plant-derived alpha-linolenic acid to EPA and DHA in humans, it is recommended to consider dietary supplementation in certain athletes with CD in selected cases (Tomczyk et al., 2023). Lastly, CD athletes should be aware that the molecular form of a fish oil preparation can have a significant pharmacokinetic effect on the amount of plasma EPA and DHA that can be incorporated for any given dose, with triglyceride-form fish oil having been demonstrated to incorporate EPA and DHA into plasma at ∼70% greater efficiency than ethyl ester-based formulations (Dyerberg et al., 2010), which is a particularly relevant distinction for those with impaired intestinal lipid absorption capacity. The algal-based omega-3 preparations are available as a triglyceride form and are free of concerns regarding contamination with gluten in fish products thus, they are suitable for athletes who have plant diet (Jäger et al., 2025).

#### 6.1.4 Fiber

Plant foods are the main sources of dietary fibre, with the exception of whole grains, which are also a source of fibre (Dhingra et al., 2012). For general health, the Academy of Nutrition and Dietetics recommends a fiber intake of 14 g per 1000 kcal consumed (Dahl and Stewart, 2015). Athletes with CD should stay away from gluten-containing grains (wheat, barley, rye) and can consume foods that are GF to provide fiber while focusing on GF whole grains, GF pseudo-cereals (e.g., brown rice, buckwheat, quinoa, certified GF oats, amaranth, millet, sorghum, teff), legumes, fruits/vegetables, nuts, and seeds (Theethira and Dennis, 2015). Most GFDs (especially those based on refined GFP) are low in fibre (Cardo et al., 2021), which can lead to constipation and GI symptoms often seen in CD (Ghunaim et al., 2024). In support of including GF whole grains and legumes for micronutrient density, observational cohorts have found lower intakes of calcium and iron in GFD users, which is related to lower fibre intakes (Thompson et al., 2005; Jamieson and Neufeld, 2020). Gradually increasing the daily amount of fibre over a period of several weeks can help to increase tolerance in athletes with CD (Mancin et al., 2025), and may be spread throughout the day across the meals and snacks depending on gastrointestinal symptoms, hydration status, and training requirements. In addition, good fiber [Microbiota-Accessible Carbohydrates (MACs)] supply will help maintain the diversity of microorganisms, especially when protein is high, and can help prevent the production of undesirable metabolites such as proteases in the rumen (Mancin et al., 2025). Dietary inclusion of beta-glucan (∼2 g/day), which can be done mainly by consuming certified GF oats, is a feasible dietary approach; preliminary data indicate a modest attenuation of post-exercise inflammatory responses, although these data are mainly from non-athlete, non-CD populations and should be extrapolated with caution (Mancin et al., 2025).

It is clinically crucial to note, however, that around 5-8% of patients with CD are sensitive to avenin, the prolamin fraction of oats that can elicit cross-reactive T-cell activation and sustain mucosal inflammation without gluten exposure. Therefore, clinical testing of individual oat tolerance is recommended before the regular use of GF oats, which should be done with serial tTG-IgA testing over a period of at least three months when a new period of GF oat introduction is planned (Pulido et al., 2009). Responses are, however, different for specific fibers, with some prebiotics (such as inulin/Fructo-Oligosaccharides) potentially exacerbating symptoms in low fermenters but providing benefits to others, making the type, dose, and timing of fiber individual especially for CD athletes (Mancin et al., 2025). Lastly, randomized trials setting optimal fibre targets and periodization around training/competition in athletes, particularly in those with CD, are lacking; guidance is extrapolated and should be adjusted to symptoms, training demands, and tolerance.

### 6.2 Micronutrients and targeted supplementation

#### 6.2.1 Iron

Iron is a fundamental element that shapes an athlete's capacity to perform at their peak (Kardasis et al., 2023). Iron deficiency with or without anemia is highly prevalent among athletes, affecting up to 52% of adolescent female athletes and approximately 30–50% of endurance athletes (Damian et al., 2021). While female athletes are predominantly affected (15–35%), male athletes also exhibit notable rates (5–15%) (Damian et al., 2021). This higher prevalence in female athletes reflects combined iron losses from menstruation—often heavy—together with more frequent low energy availability and restrictive diets that lower intake and bioavailability, a higher burden of CD and exercise-related gastrointestinal issues causing malabsorption or fecal blood loss; additional contributors include contraceptive-related inflammation (hepcidin-mediated) and bleeding risks seen in hypermobility-dominant sports (e.g., dance/gymnastics) (Damian et al., 2021). This issue is even more pronounced in individuals with CD, where iron deficiency anemia can reach up to 82% (Talarico et al., 2021). Unfortunately, even with strict adherence to a GFD, only about half of CD patients fully restore iron levels within one year, largely due to persistent intestinal inflammation and malabsorption (Annibale et al., 2001).

Mild iron deficiency (without anemia) has a significant impact on endurance performance; for instance, female university rowers with low iron stores (serum ferritin <20 μg/L) ran 21s slower in a 2 km time trial (DellaValle and Haas, 2011). Furthermore, the following complications add to the iron status, as iron absorption is lowered by 30-50% due to increased hepcidin, a hormone, which remains elevated for up to 6 h after training (Wasilewska et al., 2025). This emphasizes the need to time the intake of iron carefully around training times. In addition, athletes should have ferritin levels greater than 50 ng/mL before going to high altitudes, as there is higher demand for iron in the production of red blood cells at hypoxia (Solberg and Reikvam, 2023). The daily iron requirement for men is 8 Milligrams (mg) and for women is 18 mg (Standing Committee on the Scientific Evaluation of Dietary Reference IntakesSubcommittee of InterpretationUses of Dietary Reference Intakes, 2002), but these may not be adequate for athletes, especially for women with CD, who have low iron absorption. Studies indicate that athletes may require around 1.3 to 1.7 times the RDA of iron, which means they tend to require about 11 to 14 mg/day for men and 23 to 30 mg/day for women, making it likely that they already have higher requirements if they are currently iron deficient (Solberg and Reikvam, 2023; Nunes et al., 2018). The implications of hepcidin kinetics for CD athletes are not just about understanding that iron absorption is inhibited after exercise; they are about when and how iron is best absorbed. In a study that measured fractional iron absorption in trained runners, a long run session elevated plasma hepcidin by 51% and reduced iron absorption by 36% 2 h after exercise, demonstrating that the post-exercise window is indeed not ideal for iron absorption (Barney et al., 2022). Importantly, this research found that there was a window of increased absorption – both before and after exercise – that CD athletes and their sports support teams could apply in the real world of pre- or post-session nutrition strategies (Peeling and McKay, 2023). From a practical perspective, it is important to time iron-rich meals or supplements to occur at the start of the day, when the naturally occurring hepcidin concentrations are lowest, and to deliberately avoid the 3–6 h after exercise, which is when hepcidin concentrations are highest (Nolte et al., 2024). For CD athletes, whose intestinal absorptive capacity is already reduced, this optimization timing is more than just a strategy; it could be the very thing between being adequate and having clinically significant levels of iron deficiency.

For periods of intense training, such as altitude training, supplementation with elemental iron (100-200 mg/day) may be needed, but only under medical supervision (Stellingwerff et al., 2019). In instances of proven deficiency or anemia, advanced formulation of iron (sucrosomial iron) is preferred because it is absorbed better and has fewer gastrointestinal side effects than conventional forms of iron (e.g., ferrous sulfate) (Elli et al., 2018). Athletes with CD are encouraged to regularly consume naturally GF, iron-rich foods, including lean red meats, dark leafy greens, legumes, quinoa, amaranth, and buckwheat (Caeiro et al., 2022; Theethira and Dennis, 2015). In combination, plant-based (non-heme) iron sources with vitamin C-rich foods (citrus fruits, peppers, tomatoes) are very effective to absorb the iron (Solberg and Reikvam, 2023; Annibale et al., 2001). However, foods that are rich in calcium, coffee, and tea are advised to be avoided at the same time as foods rich in iron, since they hinder the absorption of iron (Lane and Richardson, 2014).

#### 6.2.2 Vitamin D

Vitamin D (cholecalciferol, Vitamin D3) is crucial for athletes' health, performance, and injury rehabilitation (de La Puente Yagüe et al., 2020). Despite its importance, deficiency remains common, affecting approximately 50–90% of athletes (Ghazzawi et al., 2023), particularly those training indoors (e.g., gymnastics, dance), residing at higher latitudes (>35° N/S), or having darker skin pigmentation, especially during winter (de et al., 2020; Kawashima et al., 2021). Similarly, around 8–88% of individuals with CD experience vitamin D deficiency due to impaired intestinal absorption and chronic villous atrophy (Caruso et al., 2013), with about 30% remaining deficient despite adherence to a GFD for two years (Jivraj et al., 2022). Subclinical vitamin D deficiency (25(OH)D < 30 ng/mL or 75 nmol/L) is a major risk factor for stress fracture by reducing bone strength under normal physiological stress. The risk of fifth metatarsal fracture has been reported to be 23.3 higher at levels <75.8 nmol/L (Knechtle et al., 2021). Further, athletes with low levels of vitamin D have about 12% more stress fractures than those with adequate levels, suggesting that optimal vitamin D status may help prevent stress fractures (Yoon et al., 2021).

Vitamin D also has a beneficial impact on muscle function, leading to type II muscle fibre growth, and improvements in neuromuscular performance, isometric strength (+18.7%), vertical jump (+7.1%), anaerobic power, aerobic capacity (VO_2_ max), erythropoiesis and cardiovascular efficiency (de et al., 2020; Książek et al., 2019; Wyatt et al., 2024). As part of winter training, vitamin D supplementation could improve immunity and help to limit upper respiratory infections (Jung et al., 2018). For athletes, particularly those suffering from CD, the current RDA (600–800 IU/day) may be inadequate, as it is associated with several factors that interfere with intestinal vitamin D absorption, such as consumption of certain dietary supplements, sunscreen application, wearing protective clothing, environmental pollution, and genetic factors (de et al., 2020). A study revealed that calcium and vitamin D supplementation (1000 mg calcium and 400 IU vitamin D per day) for 2 years increased bone mass in celiac patients, but the concentration did not reach that of healthy patients (Rondanelli et al., 2019). Recent guidelines suggest the serum 25(OH)D level should be kept between 30 and 50 ng/mL (75-125 nmol/L) and that concentrations exceeding 150 nmol/L should be avoided, in order to prevent toxicity (de et al., 2020; Yoon et al., 2021). For adults, >1000 IU/day has been suggested for CD-focused guidance, with maintenance being continued at this dose following the initial treatment period of 50,000 IU/week for 4-8 weeks (Lebovits and Lee, 2023). Recommendations for CD athletes may be based on a higher range of 2000–6000 IU/day, as these groups are exposed to higher musculoskeletal loading, are exposed to sunlight indoors, and have potential for malabsorption. (de et al., 2020). When vitamin D is supplemented in combination with calcium, magnesium, and vitamin K2 this has a more positive effect, aids bone metabolism, and minimizes potential side effects (Capozzi et al., 2020).

Vitamin D supplementation should include vitamin D-rich foods (e.g., fatty fish, fortified dairy, egg yolk) for athletes with CD, but this approach alone may not be enough to alleviate the deficiency. There is emerging evidence that individualized supplementation based on polymorphisms in the vitamin D-binding protein gene (GC; particularly rs4588 and rs7041) and vitamin D receptor gene (VDR; particularly FokI and BsmI variants) can significantly influence both the bioavailability of vitamin D and its responsiveness in target tissue. Residual 25(OH)D gaps may need to be significantly greater in athletes with certain high-risk haplotype combinations compared to those with the wild-type combinations to reach similar serum 25(OH)D levels (Wang et al., 2022). This inter-individual difference re-emphasizes the need for monitoring first, fixed-dose protocols not first: aiming for serum 25(OH)D levels that have been documented rather than a standard, blanket approach.

#### 6.2.3 Magnesium

Although Magnesium (Mg) is a crucial nutrient in biological processes, it is paradoxically underutilized in the fields of clinical and sports nutrition (Fatima et al., 2024). Mg is a cofactor in more than 600 enzymatic reactions and plays a role in a wide array of physiological functions, such as the regulation of glucose, calcium handling processes, oxidative phosphorylation, and neuromuscular excitability, all of which are especially important during the metabolic stress of exercise (Zhang et al., 2017). There has been an increasing incidence of Mg deficiency, largely because of dietary consumption of refined/ultra-processed foods, Mg depletion from crops, increased dietary requirements with high levels of phosphorus and Vitamin-D, losses from common medications and disease, losses due to sweat during physical activity, and because serum Mg is a poor indicator of body Mg status (DiNicolantonio et al., 2018). This deficiency is no longer limited to the general population, but has also been reported in sports populations (Zhang et al., 2023) and even in those with CD (Kreutz et al., 2020), who are even more susceptible to this deficiency because of compromised intestinal absorption and the low Mg content of many GFDs (Shepherd et al., 2017; Vici et al., 2016; Cardo et al., 2021). During physical activity, there is dynamic redistribution between the extracellular fluid and active tissues to compensate for the increased demand for Mg inside the cells (Tarsitano et al., 2024). This redistribution and lasting losses due to sweat and urine can cause transient hypomagnesemia (Zhang et al., 2023; Tarsitano et al., 2024). If these changes are not compensated for, they can disrupt Mg-adenosine triphosphate (ATP) complex formation, calcium regulation, and eventually decrease the efficiency of contraction (Zhang et al., 2017; Tarsitano et al., 2024). Deficiency can present clinically as early fatigue, muscle cramps, and slower recovery, and mechanistically has been associated with higher lactate levels and impaired glucose metabolism (Zhang et al., 2017; Tarsitano et al., 2024). For athletes, the current RDA of 400–420 mg/day for men and 310–320 mg/day for women is probably not enough, especially during periods of high training or competition (Tarsitano et al., 2024). There is a growing body of literature describing the need to increase the recommended daily intake of magnesium (RDI) by 10–20% above the RDI in response to heightened physiological demands, with the supplemental dose needed to be around 500 mg per day in many athletic settings (Tarsitano et al., 2024). The results of human studies have been mixed, however, and the benefits of Mg supplementation may be reliant on the individual's initial Mg status, type of exercise, and precision of dosage (Zhang et al., 2017). This is especially important for women athletes, where magnesium levels can vary throughout the menstrual cycle, dropping significantly during the follicular phase (when athletes are seen as being more prone to cramps, fatigue, and delayed recovery) (Tarsitano et al., 2024).

Another level of the question is the type of magnesium supplement used, since there are significant differences in the absorption rate of various compounds. The most popular and affordable form, magnesium oxide, has the lowest bioavailability, at 4%, while magnesium citrate and magnesium glycinate are organic forms that are far more bioavailable and better tolerated in people with gastrointestinal sensitivity, which is associated with the compromised mucosal barrier found in active CD (Fatima et al., 2024). This distinction is relevant to the real world for CD athletes: a poorly absorbed form can cause a good lab result but leave the intracellular and muscular stores of magnesium truly depleted, as serum magnesium (which accounts for less than 1% of total body stores) has been known to be a poor reflection of the functional magnesium status (Witkowski et al., 2012). Organic forms of magnesium administered in divided doses could be a sound and evidence-based strategy until randomized trials are conducted to determine the best formulation for this population, but especially for those with gastrointestinal sensitivity. Dietary recommendations include promoting GF foods and foods rich in magnesium (leafy green vegetables, nuts, cocoa, legumes, and pseudo-cereals such as quinoa and buckwheat) (Caeiro et al., 2022). However, it remains unclear what form of Mg is optimal for improving athletic performance, and it is important to select this type individually, depending on gastrointestinal tolerance and clinical context. The ideal time for Mg supplementation is to coincide with the window of highest plasma availability (2-2.5 h after intake), before exercise (Tarsitano et al., 2024).

#### 6.2.4 Calcium

Calcium (Ca) is a vital mineral, particularly for athletes seeking optimized performance (Ghazzawi et al., 2023). Yet, it is not uncommon for athletes to fail to meet dietary intake recommendations for Ca, with estimates ranging from 30 to 50% in endurance athletes specifically (Moss et al., 2023). Moreover, individuals with CD often suffer from Ca deficiency (41% at diagnosis) (Rondanelli et al., 2019), most likely due to the pathophysiological implications of CD (Kreutz et al., 2020), the nutritional quality of GF products lacking Ca fortification (Herrera-Quintana et al., 2025), and the prevalence of inadequate Ca consumption among individuals on a GFD (over 50%) (Rondanelli et al., 2019). Study shows that after one year of a strict GFD, bone mineral density improves significantly in adults with CD; nevertheless, because a subset remains with low Bone Mineral Density (BMD), emphasis on adequate dietary Ca and vitamin D within the GFD is warranted to support bone recovery (Pantaleoni et al., 2014).

Consequently, osteoporosis poses a risk to athletes due to increased Ca depletion (potential losses via perspiration and bone mineralization) alongside inadequate Ca intake (Ghazzawi et al., 2023). Ca also plays a fundamental role in bone health, skeletal and cardiac muscle contractions, energy metabolism, mineral density, and nerve function, all of which contribute to performance enhancement (Ghazzawi et al., 2023). Additionally, Ca aids in delaying the onset of muscle soreness, reducing fatigue, injuries, and stress fractures, as well as improving recovery time (Ghazzawi et al., 2023). Therefore, maintaining adequate calcium intake through diet, and supplementation when indicated is an important consideration. Current recommendations suggest 1500 mg/day for athletes and individuals with CD (Ghazzawi et al., 2023; Rondanelli et al., 2019; Grabia et al., 2024). Some nutrients augment Ca absorption, such as vitamin D and lactose, while high phytic acid, oxalic acid, phosphorus, and fiber can inhibit its absorption (Grabia et al., 2024)."

In addition, intestinal Ca absorption is limited to 500 mg at once, necessitating intake distribution throughout the day (Grabia et al., 2024). Calcium-rich foods that may be incorporated into the diet include dairy products, GF fortified products such as cereals, nuts and seeds (e.g., almonds, sesame, and chia), dark leafy greens (e.g., kale and watercress), and fish such as sardines and salmon (Ghazzawi et al., 2023; Cormick et al., 2019). For Ca supplementation, gluconate, citrate, and Ca carbonate are appropriate forms (Grabia et al., 2024). Altogether, regular follow-up with health professionals and individualized planning are important for athletes with CD to optimize performance.

#### 6.2.5 Zinc

Zinc is an essential trace element required for immune function, protein and nucleic-acid synthesis, wound healing, and cell division, acting as a cofactor for more than 300 enzymes (King et al., 2016). Deficiency is reported both in athletes and in individuals with CD. In CD, zinc deficiency is common, affecting approximately 60-67% of patients at diagnosis (Kreutz et al., 2020; Bledsoe et al., 2019). The underlying mechanisms include impaired absorption due to proximal small-bowel enteropathy, increased fecal losses, reduced dietary intake, low serum protein-binding capacity, and epithelial losses through damaged mucosa (Fathi et al., 2013). A study of 221 found that 48.3% of CD patients had zinc deficiency, and the prevalence among non-celiac controls on a GFD was comparable, indicating that the low intake of zinc is not related to malabsorption, but to the nutritional restrictions of a GFD (Jivraj et al., 2022). This is primarily due to the low zinc content in many GF foods and low dietary diversity (Larretxi et al., 2019).

Endurance sports are also a risk factor for zinc deficiency, and athletes with CD are especially susceptible to this micronutrient deficiency (Baranauskas et al., 2015; Micheletti et al., 2001). Systematic evidence indicates that athletes often have lower concentrations of zinc compared to non-athletes despite higher self-reported intakes, suggesting that training-induced sweating and increased physiological stress may raise zinc requirements beyond normal recommendations (Chu et al., 2018; DeRuisseau et al., 2002). In athletes, zinc deficiency may result in loss of appetite, unplanned weight loss, fatigue, and decreased endurance, and may lead to osteoporosis (Micheletti et al., 2001). The RDA is 11 mg/day for men and 8 mg/day for women (King et al., 2016). The normal range of zinc in serum or plasma is generally quoted as 80–120 μg/dL (12–18 μmol/L), and deficiency is considered at the lower limit of the range (suggested <70 μg/dL in women, and <74 μg/dL in men, although laboratory-dependent) (King et al., 2016). However, zinc status assessment could be underestimated when the assessment was performed shortly after strenuous exercise because training-induced inflammation could lower zinc transiently (Chu et al., 2017). Routine intakes of RDA should be used only if a documented deficiency is present; for athletes, the needs may be higher due to the training load, sweat losses, and potential restriction of GFD, and thus should be evaluated on an individual basis (Larson-Meyer et al., 2018).

The benefits of zinc supplementation on performance are inconsistent and inconclusive: some short-term trials have shown improvement in endurance, strength, or hematological parameters, but results have been conflicting and not conclusive (Ghazzawi et al., 2023; Heffernan et al., 2019). A food-first approach should be focused on consuming zinc-rich, GF foods, including lean meat, poultry, fish and selected shellfish (e.g., oysters, shrimp), eggs, dairy products, legumes, nuts, seeds, and pseudo-cereals (Caeiro et al., 2022; Devarshi et al., 2024). Soaking and fermentation of whole grains and legumes will increase the zinc bioavailability since phytate chelates zinc, and adding animal protein to mixed meals increases the absorption of zinc (Devarshi et al., 2024). Patients with CD may be supplemented with 25–40 mg/day of zinc until normalization, for up to three months, then every 1–2 years as needed (Rondanelli et al., 2019). Chronic intake up to the tolerable upper limit of 40 mg/day of elemental zinc is generally considered safe; however, doses ≥60 mg/day can interfere with copper absorption and should only be used under medical supervision, with periodic monitoring of copper status (Devarshi et al., 2024).

#### 6.2.6 B Vitamins

B-vitamins play a crucial role in various metabolic functions such as carbohydrate and fat oxidation, production of energy by mitochondria, synthesis of neurotransmitters, and formation of red blood cells (Ghazzawi et al., 2023). These functions work together to be a major factor in determining adaptation to training and performance in competition (Woolf and Manore, 2006). CD, ∼10–85% folate and ∼5–40% B12 are reported in patients at diagnosis and persist on long-term GFD (≈0–20% folate, ≈2.9–41% B12) (Rondanelli et al., 2019). These gaps likely reflect persistent villous atrophy/incomplete mucosal recovery in some adults and the comparatively low B-vitamin content and limited folic acid enrichment of many GFP (Rondanelli et al., 2019). Subclinical folate and B12 deficiencies are clinically relevant because of their association with hyper homocysteinemia, a biomarker linked to increased vascular risk, which may also have implications for recovery in athletes (Dickey et al., 2008).

In athletes, Thiamin (vitamin B1) is required as Thiamin Pyrophosphate (TPP) in Pyruvate Dehydrogenase (PDH) and the Tricarboxylic Acid (TCA) cycle, as well as for Branched-Chain Amino Acid (BCAA) metabolism; deficiency impairs carbohydrate oxidation, leads to lactate accumulation, and reduces exercise performance, with metabolic depletion studies confirming these effects (Heffernan et al., 2019). Although most athletes meet the RDA (1.1–1.2 mg/day), marginal deficiencies may still occur, particularly with energy restriction, and can compromise high-intensity performance. Riboflavin (vitamin B2) functions as Flavin Adenine Dinucleotide (FAD) and Flavin Mononucleotide (FMN) in electron transfer and in the activation of vitamin B6 (Heffernan et al., 2019). Evidence from metabolic studies shows that exercise and dieting increase riboflavin requirements, especially in active women, with intakes of up to 1.4 mg/1000 kcal sometimes necessary to maintain adequacy (Heffernan et al., 2019). Depletion studies demonstrate that combined low intakes of vitamin B1, vitamin B2, and vitamin B6 reduce exercise performance and elevate lactate levels (Heffernan et al., 2019). Vitamin B6, in its active form, Pyridoxal Phosphate (PLP), is essential for amino acid metabolism and glycogen breakdown, and its requirement is closely tied to protein intake, estimated at ∼0.016 mg per gram of protein (Heffernan et al., 2019). Higher protein diets and exercise increase turnover and urinary losses of 4-pyridoxic acid (Heffernan et al., 2019). Athletes with poor status have been documented to be 5–60%, with some requiring 2.0–3.0 mg/day (∼1.5–2.5 × RDA) to be adequate (Heffernan et al., 2019). Folate (vitamin B9) plays a key role in DNA synthesis, red blood cell formation, and tissue repair (Heffernan et al., 2019). Low intakes are found in female athletes and in some male athletes with low energy intake (Heffernan et al., 2019). Folate works with vitamin B12 and vitamin B6 to regulate the metabolism of methionine, and folate deficiency increases homocysteine levels (Heffernan et al., 2019). Vitamin B12 (cobalamin) plays a vital role in DNA synthesis and is important for neural integrity, and most cases of vitamin B12 deficiency result from impaired absorption (low intrinsic factor or gastric acid) (Heffernan et al., 2019). It is recognized that there is a higher risk in these groups – vegetarians and athletes excluding animal products (Heffernan et al., 2019). While there is no evidence of increased requirements with training, it is important that there is sufficient provision for energy metabolism, erythropoiesis, and long-term health (Krzywański et al., 2020).

Lean meat, fish, eggs, dairy, legumes, nuts and seeds, gluten-free pseudo-cereals, and fortified foods are mainstay food-first strategies (Caeiro et al., 2022). Athletes with CD who limit energy intake, have poor dietary habits, or do not consume animal products may benefit from a daily GF multivitamin/mineral supplement containing ∼100% of the Dietary Reference Intakes (DRIs), after correcting for folate and B vitamin deficiency (Lebovits and Lee, 2023). Specific supplementation should be considered if deficiencies are confirmed (Rondanelli et al., 2019). A dose of 500 μg to 1000 μg of vitamin B12 is effective for oral or sublingual administration; intramuscular vitamin B12 (1000 μg) should be used for severe deficiency or absorption problems (Rondanelli et al., 2019). In folate, a 3-month course of 1 mg/day of folic acid is suggested before starting maintenance therapy, which is 400–800 μg/day (Rondanelli et al., 2019). These protocols restore biomarkers, reduce homocysteine, and complete sports nutrition objectives by maintaining the ability for erythropoiesis, oxygen transport, and recovery.

#### 6.2.7 Probiotics

Probiotic supplementation has become a popular trend in recent years, as it has several potential health benefits, and its potential protective effect against the nutritional and inflammatory challenges associated with CD is emerging (Sarita et al., 2025; Mozafarybazargany et al., 2023; Francavilla et al., 2019). Around one-third of people with CD self-prescribed probiotics to help control their ongoing gastrointestinal issues, demonstrating a rise in interest in probiotics as a complementary therapy to a GFD (Joelson et al., 2021). Beneficial taxa have recently been shown to remain depleted in CD even while following a GFD, with implications for intestinal barrier integrity and nutrient absorption (Kaliciak et al., 2022). This is because dysbiosis in the gastrointestinal system can play a crucial role in affecting athletes' performance, with nutrient metabolism, energy balance, and barrier function being the most important functions (Ghaffar et al., 2024). Certain probiotic strains, particularly within the Lactobacillus and Bifidobacterium genera, produce enzymes capable of degrading immunogenic gluten fragments, including the 33-mer gliadin peptide, potentially offering additional protection against incidental gluten exposure (Leszczyńska et al., 2024; Moawad et al., 2023). These effects are strain-specific; not all strains of Lactobacillus or Bifidobacterium are equally able to induce degradation of the immunotoxic 33-mer gliadin peptide. Currently, *Bifidobacterium breve* and *Bifidobacterium longum* have shown the ability to diminish pro-inflammatory cytokines such as Tumor Necrosis Factor-alpha (TNF-α) and provoke secretion of the anti-inflammatory cytokine, IL-10, in a meaningful way to complement the process of mucosal repair while following GFD (Jedwab et al., 2022). Additionally, Al-Tanjy et al. (2025) reported that 8-week multi-strain probiotic use in CD patients improved gastrointestinal symptoms and modulated autophagy markers (atg7, atg16l1). A 6-week multi-strain study of *B. breve*, *B. animalis* subsp. lactis, *L. casei*, and *L. plantarum* demonstrated measurable improvements in gastrointestinal symptoms and microbial diversity in adults when improvements in the GFD were made with these strains compared to GFD alone, but not when just GFD was used (Laterza et al., 2025).

Multi-strain probiotic formulations should be considered as an optional treatment for athletes with gastrointestinal symptoms despite dietary management, although there is still limited and preliminary evidence and strain-specificity for routine use as a standard part of care. In one study, the use of probiotic enzymes has been shown to help increase gluten tolerance in CD patients without any effect on the markers of intestinal or serological markers (Matera and Guandalini, 2024). While these findings indicate that probiotic pre-treatment could be used to counteract gluten toxicity, it is not used to replace strict GFD following and is only recommended as a secondary precautionary measure to protect the athlete from incidental gluten exposure in the event that he or she travels or eats from untrusted restaurants. In addition, probiotics could enhance the intestinal barrier function, which is a common issue in CD patients and endurance athletes, where up to 90% of cases suffer from gut symptoms from mucosal injury and impaired blood flow to the gut (Ribichini et al., 2023). A meta-analysis in CD patients revealed a greater reduction in gastrointestinal symptoms in the probiotics group compared to the placebo group (29%) (Seiler et al., 2020). In isolation, a meta-analysis of randomized trials in various populations revealed that probiotics reduced serum zonulin, a parameter associated with gut-barrier integrity in CD, but data on CD-specific biomarkers are limited (Zheng et al., 2023). Probiotics also promoted beneficial gut microbes, such as an increase in fecal Bifidobacteria, consistent with the barrier-supporting effects (Mozafarybazargany et al., 2023). Moreover, probiotics have been shown to modulate the immune responses by stimulating production of anti-inflammatory cytokines, such as IL-10 and IL-22, and decreasing pro-inflammatory cytokines, including IL-6 and TNF-α (Matera and Guandalini, 2024; Eslami et al., 2020; Łagowska and Bajerska, 2021). The influence of probiotics on the incidence and/or duration of URTIs in athletes is limited and is highly strain-dependent (Łagowska and Bajerska, 2021; Walsh, 2019), and meta-analysis evidence does not suggest a significant difference in the incidence or severity of URTIs at the class level. This specific strain is especially crucial for athletes with CD, as those who already show residual immune dysregulation on GFD are unlikely to consistently derive benefit from probiotics with immune support rather than evidence-based immune support for specific strains.

In the micronutrients and probiotics relationship, serum ferritin improved with probiotic use (+2.45 ng/mL, p = 0.009) (Apte et al., 2025), and limited evidence was found showing that folate, B12, calcium, and zinc absorption improved in probiotics supplementation (*L. plantarum* 299v, galacto-oligosaccharides) (Barkhidarian et al., 2021). Additionally, some probiotic strains, such as *L. acidophilus*, *L. plantarum* TK10, and *L. plantarum* PS128, have been proposed to have ergogenic properties (de et al., 2023; Di Dio et al., 2023), but there is a need for more evidence and consistency. Based on the methodological constraints, more in-depth research studies, particularly with athletes who have CD, are needed. Hence, probiotics should be used as a complementary treatment to improve QOL and health in celiac patients (Ali et al., 2022), but not as a main treatment. Although there is currently robust evidence that probiotics work well as an adjunct to a comprehensive CD program, future trials of quality probiotics may help to elucidate the most effective probiotic strains and dosages.

#### 6.2.8 Glutamine

Glutamine supplementation can be beneficial in the case of athletes suffering from CD, to address gastrointestinal health challenges (Kim and Kim, 2017). Glutamine peptides can strengthen the gut barrier by supplying the intestinal epithelial cells (IEC) with the necessary energy to maintain barrier function (Wang et al., 2025). In clinical studies, oral glutamine (15 g/day for 8 weeks) significantly decreased galactosugar permeability from 0.11 to 0.05 (p < 0.0001) in patients with post-infectious irritable bowel syndrome, which clearly means that it improved barrier function and clinical symptoms of the intestine (Zhou et al., 2019). These are confirmed by mechanistic studies, which indicate that supplementation with glutamine stimulates expression of tight junction proteins and promotes mucosal healing (Kim and Kim, 2017). This is also supported by recent randomized controlled trials in gastrointestinal disorders showing the soothing effects of glutamine in cohesively restoring epithelial health by reducing levels of zonulin, an indicator of gut permeability (Shariatpanahi et al., 2019). In animal models, the diet enriched with glutamine also resulted in faster healing of damaged intestinal mucosa (Swaid et al., 2013). Furthermore, peptides derived from glutamine, like Alanyl-Glutamine (Ala-Gln), show greater stability and bioavailability than free glutamine, and they have demonstrated the ability to promote the integrity of the intestinal barrier by regulating tight junctions, mucin expression, and gut microbiota composition (Wang et al., 2025). As far as immunity is concerned, there are limited data indicating that glutamine may reduce exercise-induced immunosuppression (Song et al., 2015). One study showed that after training, glutamine supplementation lowered the rate of infection among the endurance athletes: 81% in the glutamine group were infection-free versus 49% in the placebo group (Castell et al., 1996). Nevertheless, systematic reviews and meta-analyses of the past few years have shown that the supplementation of glutamine does not uniformly impact immune cell counts, incidence of illness, aerobic performance, and body composition in the athlete population (Ahmadi et al., 2019).

Glutamine may be more important in athletes with CD because there is a barrier to mucosal integrity and reduced nutrient absorption. Plasma glutamine is decreased in active CD patients (7% decreased compared with healthy controls), and decreased plasma glutamine is associated with the degree of intestinal damage and villous atrophy (Sevinc et al., 2016).

From this point of view, the dipeptide form, Ala-Gln, is particularly interesting to consider in the context of CD athletes. Ala-Gln is structurally stable, and it is not only not degraded under the acidic conditions of the stomach, but it is also not readily broken down by digestive enzymes during digestion; instead, it is broken down specifically and efficiently upon absorption by intestinal epithelial cells to release both amino acids, Ala and Gln, in the form of free amino acids (Wang et al., 2025). When tested in an animal model of exercise-induced intestinal damage, Ala-Gln maintained mucosal integrity following acute exhaustive exercise, a test condition that resembles the gastrointestinal stress that occurs with long-duration exercise, and which significantly upregulates the expression of the tight junction proteins that are most consistently dysregulated in CD, namely claudin and occludin (Freitas et al., 2020). The dual relevance – namely, the structural vulnerability caused by CD pathology and the functional stress brought about by high-intensity training – makes Ala-Gln a compound of theoretical interest in this population, albeit no published trial has specifically tested this compound in CD athletes, and its clinical recommendation is extrapolated from the mechanistic and adjacent-population data. The practical suggestions summarized in Table 1 summarize the evidence in the literature and should not be read as formal guideline recommendations, especially when evidence is not directly from sport participants.

## 7 Assessment and monitoring of nutritional status among CD athletes

### 7.1 Diagnostic confirmation and serological monitoring

CD is often under-recognized in sport and commonly presents with non-specific, performance-limiting symptoms rather than classic gastrointestinal manifestations (Singh and Makharia, 2014). Clinicians should proactively request celiac-specific serology in athletes presenting with persistent unexplained fatigue, recurrent upper respiratory infections, iron deficiency anemia unresponsive to standard supplementation, stress fractures disproportionate to training load, unexplained performance plateau or regression, or growth faltering in adolescent athletes—recognizing that classical gastrointestinal manifestations may be absent in up to 50% of atypical CD presentations, as is frequently the case in athletic populations (Singh and Makharia, 2014; Mancini et al., 2011). Serum Tissue Transglutaminase Immunoglobulin A (tTG-IgA) is the preferred screening assay, accompanied by total Immunoglobulin A (IgA) to detect IgA deficiency (Williams et al., 2022). In confirmed deficiency, Immunoglobulin G (IgG)-based testing (tTG-IgG and/or Deamidated Gliadin Peptide IgG [DGP-IgG]) is indicated, with Endomysial Antibody (EMA) reserved as confirmatory (Williams et al., 2022). Serologic titers are lower after a strict GFD within one year; however, normalization may take longer if initial titers are high; serology is not predictive of mucosal healing (Pinto-Sanchez and Bai, 2019; Gidrewicz et al., 2017). Ensuring accuracy is more reliably achieved using serial testing with the same assay, whilst duodenal biopsy should be considered for non-response or seropositivity for >24 months after initiating a GFD (Elli et al., 2024).

HLA-DQ2/DQ8 genotyping is also useful in the context of gluten restriction in athletes, when CD serology is inconclusive, a seronegative CD is suspected, or when testing is conducted after the athlete has eliminated gluten (Lis et al., 2015; Husby et al., 2019). The absence of both alleles is negative predictive value (NPV > 99%), while the presence of one allele is not diagnostic (Husby et al., 2019). Gluten Immunogenic Peptide (GIP) testing can be used to identify recent exposure and is most useful when traveling with a team, when there are catered events, or in other high-risk scenarios; urinary GIP is usually detectable 6-48 h after exposure, whilst stool GIP is detectable 1-3 days after exposure and is more sensitive to lower-level exposures. GIP testing should be combined with serology or dietitian-led assessment, but should not be used as the sole assessment tool (Pinto-Sanchez and Bai, 2019). Intestinal fatty acid-binding protein (I-FABP) is a cytosolic protein that is released solely into the circulation when the enterocyte is damaged, and is an emerging biomarker with great potential relevance for CD athletes. Acute mucosal injury from gluten and exercise-induced intestinal ischemia is known to cause an increase in serum I-FABP, and the increase is rapid; serum I-FABP might be a more sensitive and timely marker of acute epithelial damage than tTG-IgA serology, which is a marker of cumulative rather than acute exposure. I-FABP is therefore a highly interesting research biomarker for CD, both in relation to the CD pathology itself, as well as in relation to exercise physiology, and its incremental incorporation in clinical monitoring panels should be considered (Adriaanse et al., 2013).

If diagnosed, nutritional assessment should be directed towards iron status and micronutrient status during training-related inflammation. Iron deficiency (with or without anemia) is widespread, with a normal Hb level usually becoming normal within 6–12 months on a GFD, but normal iron stores may take up to 24 months (Talarico et al., 2021). Ferritin continues to be the most important indicator of iron stores: <15 μg/L is a sign of iron deficiency, 15-30 μg/L is a sign of iron deficiency, and ≥50 μg/L is a recommended threshold before considering altitude training in athletes aged ≥15 years (Solberg and Reikvam, 2023; Clénin et al., 2015). In cases where ferritin may be confounded by heavy training or systemic inflammation, use of Soluble Transferrin Receptor (sTfR), Reticulocyte Hemoglobin Content (CHr/Ret-He), or sTfR/log ferritin should be considered in addition to the standard iron indices, and in combination with hs-CRP (Dignass et al., 2015; El-Gendy et al., 2018; World Health Organization, 2020;Al-Naseem et al., 2021; Solberg and Reikvam, 2023; Clénin et al., 2015). Deficiency is supported by CHr/Ret-He <29 pg or Transferrin Saturation (TSAT) < 20%, and during inflammation, ferritin <100 μg/L is also an indicator of low stores (Al-Naseem et al., 2021). Oral iron will improve Hb levels by 2 g/dL in 3–4 weeks and replenish stores in 8–12 weeks, and therapy may be required for 3–6 months post-correction (Kılıç et al., 2022; Jimenez et al., 2015; Cohen and Powers, 2024). These timelines assume iron therapy – correction with GFD and mucosal healing takes longer. It is important to standardize the sampling of blood, such as at the same time of day, after ≥48 h rest, and with a well-hydrated subject to minimize variation from exercise (Sanchis-Gomar and Lippi, 2014).

The present literature does not pay sufficient attention to the relationship between iron status and the omega-3 index (O3i), which are both complementary biomarkers of the level of training readiness in CD athletes. Despite reporting eating fish according to dietary guidelines, fewer than 1 in 5 athletes achieve an O3i above 8% (the cut-off point for cardiovascular protection and membrane-level anti-inflammatory action) in cross-sectional studies (Heileson et al., 2025). However, in CD, with the already impaired intestinal absorption of lipid-soluble compounds, the intake of EPA and DHA compared to the incorporation into the erythrocyte membrane is likely to be even greater, and the O3i is a clinically relevant monitoring target, in addition to the standard iron and micronutrient panel (Jäger et al., 2025). Routine assessment of O3i ideally at baseline and after any intervention with omega-3s should enable the clinical team to confirm that omega-3 repletion is actually taking place at the cell level, as opposed to relying on data from dietary recall, which can significantly overestimate omega-3 absorption in this patient group. Low BMD is frequently observed in adults with CD (Lucendo and García-Manzanares, 2013). Dual-Energy X-Ray Absorptiometry (DXA) should be performed at diagnosis and repeated after 12–24 months if BMD is low or risk factors persist (Pinto-Sanchez and Bai, 2019). As adherence to strict GFD increases, BMD progressively increases, and measurable increases are observed in the first year, followed by normalization of BMD after ∼5 years (Pinto-Sanchez and Bai, 2019). The serum level of 25-hydroxyvitamin D 25(OH)D should be maintained at ≥ 30 ng/mL for bone health and performance (Płudowski et al., 2023). Review every 1–3 months; if still low or BMD decreased, repeat measurement of Parathyroid Hormone (PTH), Ca, and phosphate (Płudowski et al., 2023). The risk of fractures is reduced, and the benefits of musculoskeletal health are optimal in the range of 40-50 ng/mL, and this benefit may not increase beyond that (Ip et al., 2022).

### 7.2 Micronutrient, bone, and metabolic monitoring

Folate and B12 deficiencies occur frequently, and Red Blood Cell (RBC) folate is a measure of long-term folate status (World Health Organization, 2015). Vitamin B12 levels of 400-700 pg/mL are good for erythropoiesis, and Hb remains at a level above 700 pg/mL (Krzywański et al., 2020). Zinc and Mg deficiencies are common and are corrected within ∼12 weeks of supplementation (Barnett et al., 2016; Lutsey et al., 2018). There is no single definitive Mg test; interpretation should be done in context (Witkowski et al., 2012). The importance of nutrient interdependence, for example, vitamin D increases calcium absorption, while copper deficiency may result in the diagnosis of iron-deficiency anemia; hence the need for integrated evaluation (Myint et al., 2018). Monitoring should be done flexibly based on training phases and athlete workload and should be influenced by clinical red flags and athlete recovery status (Lebovits and Lee, 2023; Stellingwerff et al., 2019).

Sports nutrition integration is imperative. Biochemical results are put into perspective by dietitian assessment, which includes 3–7 day weighed food records and training logs. The Low Energy Availability in Females Questionnaire (LEAF-Q) and the International Olympic Committee Relative Energy Deficiency in Sport Clinical Assessment Tool 2 (IOC REDs CAT2) are screening tools used to assess for LEA (Stellingwerff et al., 2023). The use of athlete-specific instruments, such as the Athletic Disordered Eating (ADE) tool and the Eating Disorders Screen for Athletes (EDSA), can identify the risk of disordered eating (Buckley et al., 2024; Hazzard et al., 2020).

There is no exclusion of the presence of autoimmune thyroid disease and Type 1 diabetes mellitus (Type 1DM): baseline Thyroid-Stimulating Hormone (TSH) ± Anti-Thyroid Peroxidase Antibody (anti-TPO) and Glycated Hemoglobin (HbA1c) are recommended (Pinto-Sanchez and Bai, 2019). As part of baseline evaluation and follow-up, liver enzymes (AST and ALT) should be evaluated. However, it is important to be aware that athletes with CD, ALT and AST can be elevated due to skeletal muscle injury resulting from high-intensity training and may make interpretation of hepatic transaminase levels difficult. In CD athletes involved in intensive training programmes, a liver-specific marker in addition to ALT/AST is provided by the measurement of gamma-glutamyl transferase (GGT), which is not released by skeletal muscle during exercise stress (Pettersson et al., 2008). Transaminitis is common and usually improves over 6-12 months after GFD (King et al., 2020), but if it persists, it should be considered for celiac hepatitis or non-alcoholic fatty liver disease (NAFLD) that is more common in CD. The highest risk is in the first year after diagnosis, and it is high in the long term (Reilly et al., 2015).

### 7.3 Functional performance monitoring

Once nutritional assessment has been performed, functional and performance evaluation in CD athletes involves monitoring recovery and training adaptation to a GFD, and involves sport-specific testing of aerobic capacity, strength, and power as indicators of overall performance readiness. The Maximal Oxygen Uptake (VO_2_max) is used to assess aerobic efficiency and oxygen transport and could be affected in CD athletes by iron deficiency and LEA (Srivastava et al., 2024; Keller et al., 2024; Jurov et al., 2022). It is a gold standard marker of cardiovascular fitness, especially relevant for endurance sports like running, cycling, and swimming (Buttar et al., 2019). Aerobic capacity is restored (Rađen et al., 2011) following improvements after iron repletion and compliance with a GFD, but if the VO_2max_ is still suppressed, it is an indication of failure to recover. Countermovement Jump (CMJ) is a simple and reliable measure of lower body power and neuromuscular recovery (Anicic et al., 2023) and has been extensively used in sports to assess explosive performance, including athletics, football and basketball. There is evidence for adequate Vitamin D to support upper- and lower-limb strength (Tomlinson et al., 2015), protein intake of ≥1.6 g/kg/day to promote lean mass and lower-body strength (Nunes et al., 2022), and evidence that mild dehydration (∼2% body loss) impairs jump performance despite rehydration (Sekiguchi et al., 2022). In combination, inadequate Vitamin D, protein consumption, or hydration can hinder neuromuscular recovery and reduce lower extremity performance (Hwang and Yang, 2024). However, DXA is able to differentiate Fat-Free Mass (FFM) recovery from fat gain because a gain in fat mass could mask the presence of underlying sarcopenia (Shepherd et al., 2017). In a study of ≥1 year of a GFD, fat mass rises by ∼4 kg, and FFM does not recover completely (Vereczkei et al., 2021). This is a pattern of “normal-weight” obesity, commonly theorised to be linked to lower strength and metabolic efficiency (De Lorenzo et al., 2022). Regular body composition assessment is also important for optimizing performance, helping to prevent RED-S and ensuring that the body's goals are supported by the functional outcomes (Mathisen et al., 2023). Handgrip Strength (HGS) is an easy, cost-effective, and reliable measure of muscle function and physical capacity (Vaishya et al., 2024). It is a practical measure of strength, nutritional status, and physical condition of the upper limb, and is highly correlated with lean mass, total body strength, and kinetic chain performance (Pikosky et al., 2022; Cronin et al., 2017). In athletes, HGS has been found to be strongly correlated with FFM (r = 0.918, p < 0.001), which serves as the key contributor to muscle function in highly trained athletes (Di Vincenzo et al., 2020). A greater gain in FFM and HGS will be a sign of adequate protein intake and training adaptation, whereas a high gain in fat with no increase in strength will indicate a lack of training efficiency and/or nutritional and/or metabolic efficiency.

The One Repetition Maximum (1RM) test may also be used to assess maximal strength (Seo et al., 2012) for high-performance athletes. Heart Rate Variability (HRV) is a non-invasive indicator of autonomic recovery/readiness. The effect of training stress is more sensitive when combining supine and standing HRV (Dong, 2016; Maggioni et al., 2020). The quality of sleep is an overlooked recovery factor in athletes with CD; systematic review data suggest that around 61% of adults with CD experience poor sleep quality and 35% fulfill the clinical criteria for insomnia, which is much higher than the percentage generally reported in athletes without chronic disease (Beas et al., 2024). There may be multiple underlying mechanisms including nocturnal gastrointestinal disturbance, anxiety due to the CD, and, importantly, malabsorption of the amino acid tryptophan because of small bowel enteropathy. Tryptophan is the only amino acid that can serve as a dietary precursor to serotonin and melatonin, and a reduction in these neurotransmitters directly impacts sleep onset, sleep maintenance, and entrainment of the sleep-wake cycle, which each have direct implications for athletic recovery, neuromuscular readiness, and hormonal response to training (Cossu et al., 2017). Thus, screening for sleep disturbances with the ASSQ is especially indicated for this population (Samuels et al., 2016).

If fatigue, gastrointestinal upset, or failure to keep up despite normalized serology remain, more in-depth testing for persistence of malabsorption or gastrointestinal function should be considered. Lactose or fructose malabsorption, Small Intestinal Bacterial Overgrowth (SIBO), Exocrine Pancreatic Insufficiency (EPI), and Inflammatory Bowel Disease (IBD) are common causes (Rana and Malik, 2014; Tansel and Levinthal, 2023). The test should be performed away from heavy training to reduce confounders.

### 7.4 Patient-reported outcomes

Patient-Reported Outcomes (PROs) are the patients' symptoms, their perceived recovery, and their QOL (Clifford et al., 2020). The Frequency and Severity of Celiac-Related Symptoms (CSI) rating scale assesses the frequency and severity of any symptoms of the CD; the Gastrointestinal Symptom Rating Scale (GSRS) measures the intensity and impact of gastrointestinal symptoms to have an overall measure of the digestive tolerance; Fatigue Severity Scale (FSS) measures perceived fatigue and its effect on daily functioning and performance; the CD-QOL scale is a rating scale of the disease-specific psychosocial dimensions, such as the extent of any limitation and dysphoria, the health concerns, and the level of satisfaction with treatment. These instruments offer a multi-dimensional approach to maximize health, recovery, and performance outcomes in CD athletes.

## 8 Recommendations and directions for future research

To the best of our knowledge, existing reviews of CD in sport have addressed either nutritional management or clinical monitoring in isolation. The present review is among the first to integrate both domains nutritional assessment, supplementation considerations, and functional performance monitoring within a single athlete-focused framework, an approach we believe adds practical value to clinical and research settings. It brings together the scattered evidence to provide sport-specific, evidence-based advice to clinicians, sports nutritionists, and coaches, and highlights the knowledge and practice gaps. The challenge for clinical practice is to move from a generic dietetic recommendation to a sport-specific and periodized macronutrient recommendation based on the athlete's sport, training dose, and the athlete's CD-related complications. As detailed in Section 7, a standardized micronutrient and bone-health monitoring panel should be embedded in routine clinical follow-up rather than treated as a research-only exercise. Most importantly, supplements should be used only as a supplement to the GFD and should only be used if deficiencies are identified. Also, clinical protocols need to be uniform in training-aware sampling protocols, such as morning blood sampling at least 48 h after intense exercise, and hydration, and integrate the use of biomarkers with in-season performance parameters. DXA is the gold standard for body composition, and multi-frequency Bioelectrical Impedance Analysis (BIA) is a practical field method for tracking body composition frequently.

Moving forward, future research must address three primary domains to advance the care of athletes with CD.

First, high-powered RCTs using well-documented and confirmed diagnosis of CD at enrolment, with biopsies, adherence-verified GFD comparator arms, and documented baseline serological status must be prioritized in nutritional and performance trials. The factors that should be examined in sample stratification are age, sex, type of sport (endurance, power, team discipline), duration of disease, degree of current mucosal healing (Marsh grade), and training load, all of which are known to significantly affect nutritional status and performance trajectory, but are not routinely controlled for in current studies. Primary outcomes should combine objective biomarker panels with standardized functional performance testing measures to facilitate cross-study comparison and meta-analytic synthesis. These studies should focus on determining optimal nutrition programs for a variety of athletic situations, including endurance, power sports, and altitude training. Research is needed to give clear guidelines on dosage and timing of the micronutrient interventions, and to specifically correlate them with objective evaluation of athletic readiness and performance.

Second, mechanistic and translational research is much needed to elucidate the effects of strenuous exercise on intestinal barrier function in this population. Assessing markers like intestinal permeability and tight junction signatures will create a needed foundation. Data on serum levels of I-FABP, zonulin, claudin-3, and specific times before, during, and after exercise, gluten exposure, and dietary changes would provide a new source of mechanistic information that is not available in the literature. This knowledge will help to critically assess the efficacy of microbiome-based supplements (e.g., certain probiotics, alanyl-glutamine) used as a supportive therapy that can be used in conjunction with the strict GFD.

Finally, the psychosocial and infrastructure needs to be researched. Implementation effectiveness studies need to be conducted to address psychosocial stressors that are linked to anxiety and risk of RED-S, as well as practical issues like the cost of the GF diet and risk of cross-contamination. Two priorities are needed to accelerate this progress: there is a need for a validated, athlete-focused CD screening checklist and an international registry. This registry will facilitate harmonization of dietary adherence, biomarker trends, and performance outcomes over time by cohorts and allow for a model of proactive, personalized, and evidence-based management of CD athletes.

## 9 Conclusion

To conclude, the CD athlete is a population that is clinically relevant, but largely under-researched, and in which the challenges of dietary restriction, malabsorption, gastrointestinal symptoms, psychosocial burden, and performance demands converge. The present literature emphasizes the need for individualized, periodized nutritional assessment, including iron indices, 25(OH)D, calcium, magnesium, zinc, B-vitamin status, and bone mineral density monitoring, combined with functional performance assessment and context-specific nutritional planning, based on strict GFD. A single framework for monitoring and management was synthesized herein that brings these dimensions together in a single athlete-centred monitoring and management structure that, to our knowledge, has not been presented previously in the literature and is offered as a practice tool for clinicians, sports dietitians and researchers and as a methodological scaffold for the athlete-specific interventional studies that are so sorely needed in this field. However, most of this evidence is circumstantial, and there are relatively few studies that have been designed specifically in an athletic population with proven CD. In this regard, the present review should be considered as an integrative and practice-oriented framework to support clinical reasoning and to guide future prospective and interventional studies, not as a definitive guideline.

## Generative AI statement

The authors state that they did not use any generative AI tools for the scientific content of this manuscript. The text has only been edited for clarity, readability, and language using AI-assisted technologies. All revisions have been done under the direct supervision of the authors, who have carefully studied and approved all revisions.

## CRediT authorship contribution statement

Osaid Bassam Yamani: Conceptualization, Methodology, Writing - original draft.

Maha Alghamdi: Writing - original draft.

Alyaa Zagzoog: Writing - original draft, Writing - review & editing.

Esraa Noorwali: Supervision, Writing - review & editing, Visualization.

Rahaf Abdulhadi Alotaibi: Visualization.

Abdullah Alghannam: Supervision, Writing - review & editing.

Majid Almadi: Writing - review & editing, Visualization.

Khalid Aljaloud: Methodology, Writing - review & editing.

Alan Aragon: Writing - review & editing.

Nour Amin Elsahoryi: Methodology, Writing - original draft, Supervision, Writing - review & editing.

## Funding

No funding was received for the research, authorship, or publication of this article.

## Declaration of competing interest

The authors declare no known competing financial interests or personal relationships that may have influenced the work reported in this article.

## References

[bib1] Abdi F., Zuberi S., Blom J.J., Armstrong D., Pinto-Sanchez M.I. (2023). Nutritional considerations in celiac disease and non-celiac gluten/wheat sensitivity. Nutrients.

[bib2] Abdulla A., Garemo M. (2018). High cost of gluten free products might be challenging for people with celiac disease in the United Arab Emirates. Int. J. Celiac Dis..

[bib3] Abu‐Janb N., Jaana M. (2020). Facilitators and barriers to adherence to gluten‐free diet among adults with celiac disease: a systematic review. J. Hum. Nutr. Diet..

[bib4] Adriaanse M.P.M., Tack G.J., Passos V.L., Damoiseaux J.G.M.C., Schreurs M.W.J., Van Wijck K. (2013). Serum I‐FABP as marker for enterocyte damage in coeliac disease and its relation to villous atrophy and circulating autoantibodies. Aliment. Pharmacol. Ther..

[bib5] Ahmadi A.R., Rayyani E., Bahreini M., Mansoori A. (2019). The effect of glutamine supplementation on athletic performance, body composition, and immune function: a systematic review and a meta-analysis of clinical trials. Clin. Nutr..

[bib6] Al-Naseem A., Sallam A., Choudhury S., Thachil J. (2021). Iron deficiency without anaemia: a diagnosis that matters. Clin. Med..

[bib7] Al-Sunaid F.F., Al-Homidi M.M., Al-Qahtani R.M., Al-Ashwal R.A., Mudhish G.A., Hanbazaza M.A., Al-Zaben A.S. (2021). The influence of a gluten-free diet on health-related quality of life in individuals with celiac disease. BMC Gastroenterol..

[bib8] Al-Tanjy B.M., Al-Khlifeh E.M., Elsahoryi N.A., Tarawneh A., Hassanat A. (2025). Impact of probiotics on blood cell counts selected autophagy markers and improvement of symptoms in individuals with celiac disease. J. Appl. Pharmaceut. Sci..

[bib9] Al-Toma A., Volta U., Auricchio R., Castillejo G., Sanders D.S., Cellier C., Lundin K.E. (2019). European society for the study of coeliac disease (ESsCD) guideline for coeliac disease and other gluten-related disorders. United Eur. Gastroenterol. J..

[bib10] Ali B., Khan A.R., Khan A.R. (2022). Efficacy of probiotics in management of celiac disease. Cureus.

[bib11] Amawi A., AlKasasbeh W., Jaradat M., Almasri A., Alobaidi S., Hammad A.A. (2024). Athletes' nutritional demands: a narrative review of nutritional requirements. Front. Nutr..

[bib12] Anicic Z., Janicijevic D., Knezevic O.M., Garcia-Ramos A., Petrovic M.R., Cabarkapa D., Mirkov D.M. (2023). Assessment of countermovement jump: what should we report?. Life.

[bib13] Annibale B., Severi C., Chistolini A., Antonelli G., Lahner E., Marcheggiano A. (2001). Efficacy of gluten-free diet alone on recovery from iron deficiency anemia in adult celiac patients. Official journal of the American College of Gastroenterology| ACG.

[bib14] Apte A., Parge A., Nimkar R., Sinha A. (2025). Effect of probiotic and prebiotics supplementation on hemoglobin levels and iron absorption among women of reproductive age and children: a systematic review and meta-analysis. BMC nutrition.

[bib15] Araújo H.M.C., Araújo W.M.C. (2011). Coeliac disease. Following the diet and eating habits of participating individuals in the Federal District, Brazil. Appetite.

[bib16] Arias-Gastelum M., Cabrera-Chávez F., de Jesús Vergara-Jiménez M., Ontiveros N. (2018). The gluten-free diet: access and economic aspects and impact on lifestyle. Nutr. Diet. Suppl..

[bib17] Arıcı H., Tor Y.B., Altınkaynak M., Erten N., Saka B., Bayramlar O.F. (2025). Personalized diet with or without physical exercise improves nutritional status, muscle strength, physical performance, and quality of life in malnourished older adults: a prospective randomized controlled study. Nutrients.

[bib18] Aucoin M., Lalonde-Parsi M.J., Cooley K. (2014). Mindfulness‐based therapies in the treatment of functional gastrointestinal disorders: a meta‐analysis. Evid. Based Complement. Alternat. Med..

[bib19] Baethge C., Goldbeck-Wood S., Mertens S. (2019). SANRA—A scale for the quality assessment of narrative review articles. Res. Integrity Peer Rev..

[bib20] Ballestero-Fernández C., Varela-Moreiras G., Úbeda N., Alonso-Aperte E. (2021). Nutritional status in Spanish adults with celiac disease following a long-term gluten-free diet is similar to non-celiac. Nutrients.

[bib21] Baranauskas M., Stukas R., Tubelis L., Žagminas K., Šurkienė G., Švedas E. (2015). Nutritional habits among high-performance endurance athletes. Medicina.

[bib23] Barkhidarian B., Roldos L., Iskandar M.M., Saedisomeolia A., Kubow S. (2021). Probiotic supplementation and micronutrient status in healthy subjects: a systematic review of clinical trials. Nutrients.

[bib24] Barnett J.B., Dao M.C., Hamer D.H., Kandel R., Brandeis G., Wu D. (2016). Effect of zinc supplementation on serum zinc concentration and T cell proliferation in nursing home elderly: a randomized, double-blind, placebo-controlled trial. Am. J. Clin. Nutr..

[bib25] Barney D.E., Ippolito J.R., Berryman C.E., Hennigar S.R. (2022). A prolonged bout of running increases hepcidin and decreases dietary iron absorption in trained female and male runners. J. Nutr..

[bib26] Beas R., Godoy A., Norwood D.A., Ascencio Y.O., Izquierdo-Veraza D., Montalvan-Sanchez E.E. (2024). Cognitive impairment and insomnia in celiac disease: a systematic review and meta-analysis. Gut Liver.

[bib27] Black K.E., Skidmore P., Brown R.C. (2012). Case study: nutritional strategies of a cyclist with celiac disease during an ultraendurance race. Int. J. Sport Nutr. Exerc. Metabol..

[bib28] Bledsoe A.C., King K.S., Larson J.J., Snyder M., Absah I., Murray J.A. (2019).

[bib29] Bozorg S.R., Lee A.R., Mårild K., Murray J.A. (2024). The economic iceberg of celiac disease: more than the cost of gluten-free food. Gastroenterology.

[bib30] Braamskamp M.J., Dolman K.M., Tabbers M.M. (2010). Clinical practice: protein-losing enteropathy in children. Eur. J. Pediatr..

[bib31] Buckley G.L., Lassemillante A.C.M., Cooke M.B., Belski R. (2024). The development and validation of a disordered eating screening tool for current and former athletes: the athletic disordered eating (ADE) screening tool. Nutrients.

[bib32] Burden M., Mooney P.D., Blanshard R.J., White W.L., Cambray-Deakin D.R., Sanders D.S. (2015). Cost and availability of gluten-free food in the UK: in store and online. Postgrad. Med. J..

[bib33] Burger J.P., de Brouwer B., IntHout J., Wahab P.J., Tummers M., Drenth J.P. (2017). Systematic review with meta-analysis: dietary adherence influences normalization of health-related quality of life in coeliac disease. Clin. Nutr..

[bib34] Buttar K.K., Saboo N., Kacker S. (2019). A review: maximal oxygen uptake (VO2 max) and its estimation methods. International Journal of Physical Education, Sports and Health.

[bib35] Caeiro C., Pragosa C., Cruz M.C., Pereira C.D., Pereira S.G. (2022). The role of pseudocereals in celiac disease: reducing nutritional deficiencies to improve well‐being and health. J. Nutr. Metab..

[bib36] Caio G., Volta U., Sapone A., Leffler D.A., De Giorgio R., Catassi C., Fasano A. (2019). Celiac disease: a comprehensive current review. BMC Med..

[bib37] Calvo-Lerma J., Crespo-Escobar P., Martínez-Barona S., Fornés-Ferrer V., Donat E., Ribes-Koninckx C. (2019). Differences in the macronutrient and dietary fibre profile of gluten-free products as compared to their gluten-containing counterparts. Eur. J. Clin. Nutr..

[bib38] Campagna G., Pesce M., Tatangelo R., Rizzuto A., La Fratta I., Grilli A. (2017). The progression of coeliac disease: its neurological and psychiatric implications. Nutr. Res. Rev..

[bib40] Cannataro R., Abrego-Guandique D.M., Straface N., Cione E. (2024). Omega-3 and sports: focus on inflammation. Life.

[bib39] Cannataro R., Morucci G., Moni L., Malorgio M., Ryskalin L., Gesi M. (2023). Management of a high-level breaststroke swimmer with celiac disease: a case report. Curr. Sports Med. Rep..

[bib41] Cao W., He Y., Fu R., Chen Y., Yu J., He Z. (2025). A review of carbohydrate supplementation approaches and strategies for optimizing performance in elite long-distance endurance. Nutrients.

[bib42] Capozzi A., Scambia G., Lello S. (2020). Calcium, vitamin D, vitamin K2, and magnesium supplementation and skeletal health. Maturitas.

[bib43] Cardo A., Churruca I., Lasa A., Navarro V., Vázquez-Polo M., Perez-Junkera G., Larretxi I. (2021). Nutritional imbalances in adult celiac patients following a gluten-free diet. Nutrients.

[bib44] Caruso R., Pallone F., Stasi E., Romeo S., Monteleone G. (2013). Appropriate nutrient supplementation in celiac disease. Ann. Med..

[bib45] Casella G., Bordo B.M., Schalling R., Villanacci V., Salemme M., Di Bella C., Bassotti G. (2016). Neurological disorders and celiac disease. Minerva Gastroenterol. Dietol..

[bib46] Castell L.M., Newsholme E.A., Poortmans J.R. (1996). Does glutamine have a role in reducing infections in athletes?. Eur. J. Appl. Physiol. Occup. Physiol..

[bib47] Catassi C., Gatti S., Fasano A. (2014). The new epidemiology of celiac disease. J. Pediatr. Gastroenterol. Nutr..

[bib48] Cheng F.W., Handu D. (2020). Nutrition assessment, interventions, and monitoring for patients with celiac disease: an evidence analysis center scoping review. J. Acad. Nutr. Diet..

[bib49] Cherpak C.E. (2019). Mindful eating: a review of how the stress-digestion-mindfulness triad may modulate and improve gastrointestinal and digestive function. Integr. Med.: A Clinician's Journal.

[bib51] Chu A., Holdaway C., Varma T., Petocz P., Samman S. (2018). Lower serum zinc concentration despite higher dietary zinc intake in athletes: a systematic review and meta-analysis. Sports Med..

[bib50] Chu A., Petocz P., Samman S. (2017). Plasma/serum zinc status during aerobic exercise recovery: a systematic review and meta-analysis. Sports Med..

[bib52] Ciacci C., Gennarelli D., Esposito G., Tortora R., Salvatore F., Sacchetti L. (2006). Hereditary fructose intolerance and celiac disease: a novel genetic association. Clin. Gastroenterol. Hepatol..

[bib53] Cialdella-Kam L., Kulpins D., Manore M.M. (2016). Vegetarian, gluten-free, and energy restricted diets in female athletes. Sports.

[bib54] Cielo D., Galatola M., Fernandez-Jimenez N., De Leo L., Garcia-Etxebarria K., Loganes C. (2019). Combined analysis of methylation and gene expression profiles in separate compartments of small bowel mucosa identified celiac disease patients' signatures. Sci. Rep..

[bib55] Clappison E., Hadjivassiliou M., Zis P. (2020). Psychiatric manifestations of coeliac disease, a systematic review and meta-analysis. Nutrients.

[bib56] Clénin G., Cordes M., Huber A., Schumacher Y.O., Noack P., Scales J., Kriemler S. (2015). Iron deficiency in sports-definition, influence on performance and therapy. Swiss Med. Wkly..

[bib57] Clifford S., Taylor A.J., Gerber M., Devine J., Cho M., Walker R. (2020). Concepts and instruments for patient-reported outcome assessment in celiac disease: literature review and experts' perspectives. Value Health.

[bib58] Coburn S., Germone M., McGarva J., Taft T. (2022). Psychological considerations for food intolerances: celiac sprue, eosinophilic esophagitis, and non-celiac gluten sensitivity. Gastroenterol. Clin..

[bib59] Cohen C.T., Powers J.M. (2024). Nutritional strategies for managing iron deficiency in adolescents: approaches to a challenging but common problem. Adv. Nutr..

[bib60] Cohen I.S., Day A.S., Shaoul R. (2019). Gluten in celiac disease—more or less?. Rambam Maimonides medical journal.

[bib61] Cormick G., Belizán J.M. (2019). Calcium intake and health. Nutrients.

[bib62] Cossu G., Carta M.G., Contu F., Mela Q., Demelia L., Elli L., Dell'Osso B. (2017). Coeliac disease and psychiatric comorbidity: epidemiology, pathophysiological mechanisms, quality-of-life, and gluten-free diet effects. Int. Rev. Psychiatr..

[bib63] Costa A., Brito G.A.D. (2022). Aerobic exercise associated with fish oil supplementation decreases c‐reactive protein and interleukin‐6 in celiac disease patients. J. Nutr. Metab..

[bib64] Costa R.J., Snipe R.M., Kitic C.M., Gibson P.R. (2017). Systematic review: exercise‐induced gastrointestinal syndrome—implications for health and intestinal disease. Aliment. Pharmacol. Ther..

[bib65] Croall I.D., Sanders D.S., Hadjivassiliou M., Hoggard N. (2020). Cognitive deficit and white matter changes in persons with celiac disease: a population-based study. Gastroenterology.

[bib66] Cronin J., Lawton T., Harris N., Kilding A., McMaster D.T. (2017). A brief review of handgrip strength and sport performance. J. Strength Condit Res..

[bib84] D’angelo S., Cusano P., Di Palma D. (2020). Gluten-free diets in athletes. J. Phys. Educ. Sport.

[bib67] Dahl W.J., Stewart M.L. (2015). Position of the academy of nutrition and dietetics: health implications of dietary fiber. J. Acad. Nutr. Diet..

[bib68] Damian M.T., Vulturar R., Login C.C., Damian L., Chis A., Bojan A. (2021). Anemia in sports: a narrative review. Life.

[bib69] de La Puente Yagüe M., Collado Yurrita L., Ciudad Cabañas M.J., Cuadrado Cenzual M.A. (2020). Role of vitamin D in athletes and their performance: current concepts and new trends. Nutrients.

[bib70] De Lorenzo A., Pellegrini M., Gualtieri P., Itani L., El Ghoch M., Di Renzo L. (2022). The risk of sarcopenia among adults with normal-weight obesity in a nutritional management setting. Nutrients.

[bib72] DellaValle D.M., Haas J.D. (2011). Impact of iron depletion without anemia on performance in trained endurance athletes at the beginning of a training season: a study of female collegiate rowers. Int. J. Sport Nutr. Exerc. Metabol..

[bib73] Demirkesen I., Ozkaya B. (2022). Recent strategies for tackling the problems in gluten-free diet and products. Crit. Rev. Food Sci. Nutr..

[bib74] DeRuisseau K.C., Cheuvront S.N., Haymes E.M., Sharp R.G. (2002). Sweat iron and zinc losses during prolonged exercise. Int. J. Sport Nutr. Exerc. Metabol..

[bib75] Devarshi P.P., Mao Q., Grant R.W., Hazels Mitmesser S. (2024). Comparative absorption and bioavailability of various chemical forms of zinc in humans: a narrative review. Nutrients.

[bib76] Dhingra D., Michael M., Rajput H., Patil R.T. (2012). Dietary fibre in foods: a review. J. Food Sci. Technol..

[bib77] Di Dio M., Calella P., Pelullo C.P., Liguori F., Di Onofrio V., Gallè F., Liguori G. (2023). Effects of probiotic supplementation on sports performance and performance-related features in athletes: a systematic review. Int. J. Environ. Res. Publ. Health.

[bib78] Di Vincenzo O., Marra M., Morlino D., Speranza E., Sammarco R., Cioffi I., Scalfi L. (2020). Icsports.

[bib79] Dickey W., Ward M., Whittle C.R., Kelly M.T., Pentieva K., Horigan G. (2008). Homocysteine and related B-vitamin status in coeliac disease: effects of gluten exclusion and histological recovery. Scand. J. Gastroenterol..

[bib80] Dignass A.U., Gasche C., Bettenworth D., Birgegård G., Danese S., Gisbert J.P. (2015). European consensus on the diagnosis and management of iron deficiency and anaemia in inflammatory bowel diseases. J. Crohns Colitis.

[bib81] DiNicolantonio J.J., O'Keefe J.H., Wilson W. (2018). Subclinical magnesium deficiency: a principal driver of cardiovascular disease and a public health crisis. Open Heart.

[bib82] Dong J.G. (2016). The role of heart rate variability in sports physiology. Exp. Ther. Med..

[bib83] Dyerberg J., Madsen P., Møller J.M., Aardestrup I., Schmidt E.B. (2010). Bioavailability of marine n-3 fatty acid formulations. Prostagl. Leukot. Essent. Fat. Acids.

[bib85] Eberman L.E., Cleary M.A. (2005). Celiac disease in an elite female collegiate volleyball athlete: a case report. J. Athl. Train..

[bib86] El-Gendy F.M., El-Hawy M.A., Rizk M.S., El-Hefnawy S.M., Mahmoud M.Z. (2018). Value of soluble transferrin receptors and sTfR/log ferritin in the diagnosis of iron deficiency accompanied by acute infection. Indian J. Hematol. Blood Transfus..

[bib87] Elli L., Ferretti F., Branchi F., Tomba C., Lombardo V., Scricciolo A. (2018). Sucrosomial iron supplementation in anemic patients with celiac disease not tolerating oral ferrous sulfate: a prospective study. Nutrients.

[bib88] Elli L., Leffler D., Cellier C., Lebwohl B., Ciacci C., Schumann M., Sanders D.S. (2024). Guidelines for best practices in monitoring established coeliac disease in adult patients. Nat. Rev. Gastroenterol. Hepatol..

[bib90] Elsahoryi N.A., Ibrahim M.O., Alhaj O.A. (2024). Adherence to the gluten-free diet role as a mediating and moderating of the relationship between food insecurity and health-related quality of life in adults with celiac disease: cross-sectional study. Nutrients.

[bib91] Eslami M., Bahar A., Keikha M., Karbalaei M., Kobyliak N.M., Yousefi B. (2020). Probiotics function and modulation of the immune system in allergic diseases. Allergol. Immunopathol..

[bib92] Fathi F., Ektefa F., Tafazzoli M., Rostami K., Nejad M.R., Fathi M. (2013). The concentration of serum zinc in celiac patients compared to healthy subjects in Tehran. Gastroenterol. Hepatol. Bed Bench.

[bib93] Fatima G., Dzupina A., Alhmadi H.B., Magomedova A., Siddiqui Z., Mehdi A. (2024). Magnesium matters: a comprehensive review of its vital role in health and diseases. Cureus.

[bib94] Francavilla R., Piccolo M., Francavilla A., Polimeno L., Semeraro F., Cristofori F. (2019). Clinical and microbiological effect of a multispecies probiotic supplementation in celiac patients with persistent IBS-type symptoms: a randomized, double-blind, placebo-controlled, multicenter trial. J. Clin. Gastroenterol..

[bib95] Fraser C.N., Möller S.P., Knowles S.R. (2022). Understanding disease-specific and non-specific factors predicting disordered eating in adults with coeliac disease. Appetite.

[bib96] Freitas A.K.L., Silva M.T.B., Silva C.M.S., Prata M.M.G., Rodrigues F.A.P., Siqueira R.J.B. (2020). Alanyl-glutamine protects the intestinal barrier function in trained rats against the impact of acute exhaustive exercise. Braz. J. Med. Biol. Res..

[bib97] Gallant T.L., Ong L.F., Wong L., Sparks M., Wilson E., Puglisi J.L., Gerriets V.A. (2024). Low energy availability and relative energy deficiency in sport: a systematic review and meta-analysis. Sports Med..

[bib98] Ganji R., Moghbeli M., Sadeghi R., Bayat G., Ganji A. (2019). Prevalence of osteoporosis and osteopenia in men and premenopausal women with celiac disease: a systematic review. Nutr. J..

[bib99] Ghaffar T., Ubaldi F., Volpini V., Valeriani F., Romano Spica V. (2024). The role of gut microbiota in different types of physical activity and their intensity: systematic review and meta-analysis. Sports.

[bib100] Ghazzawi H.A., Hussain M.A., Raziq K.M., Alsendi K.K., Alaamer R.O., Jaradat M. (2023). Exploring the relationship between micronutrients and athletic performance: a comprehensive scientific systematic review of the literature in sports medicine. Sports.

[bib102] Ghosh S., Khetarpal P., Senapati S. (2022). Functional implications of the CpG island methylation in the pathogenesis of celiac disease. Mol. Biol. Rep..

[bib103] Ghunaim M., Seedi A., Alnuman D., Aljohani S., Aljuhani N., Almourai M. (2024). Impact of a gluten-free diet in adults with celiac disease: nutritional deficiencies and challenges. Cureus.

[bib104] Gidrewicz D., Trevenen C.L., Lyon M., Butzner J.D. (2017). Normalization time of celiac serology in children on a gluten‐free diet. J. Pediatr. Gastroenterol. Nutr..

[bib105] Gnodi E., Meneveri R., Barisani D. (2022). Celiac disease: from genetics to epigenetics. World J. Gastroenterol..

[bib106] Grabia M., Perkowski J., Socha K., Markiewicz-Żukowska R. (2024). Female athlete triad and relative energy deficiency in sport (REDs): nutritional management. Nutrients.

[bib107] Guennouni M., El Khoudri N., Bourrouhouate A., Hilali A. (2022). Availability and cost of gluten-free products in Moroccan supermarkets and e-commerce platforms. Br. Food J..

[bib108] Guest N.S., Horne J., Vanderhout S.M., El-Sohemy A. (2019). Sport nutrigenomics: personalized nutrition for athletic performance. Front. Nutr..

[bib109] Gutowski E.D., Weiten D., Green K.H., Rigaux L.N., Bernstein C.N., Graff L.A. (2020). Can individuals with celiac disease identify gluten-free foods correctly?. Clinical nutrition ESPEN.

[bib110] Hahn S.M. (2020). Food labeling; gluten-free labeling of fermented or hydrolyzed food. Fed. Regist..

[bib111] Harnett J.E., Myers S.P. (2020). Quality of life in people with ongoing symptoms of coeliac disease despite adherence to a strict gluten-free diet. Sci. Rep..

[bib112] Hart J.M., Kahanov L., Eberman L.E. (2011). Recognizing celiac disease in the athletic population. Athl. Train. Sports Health Care.

[bib113] Hazzard V.M., Schaefer L.M., Mankowski A., Carson T.L., Lipson S.M., Fendrick C. (2020). Development and validation of the Eating Disorders Screen for Athletes (EDSA): a brief screening tool for male and female athletes. Psychol. Sport Exerc..

[bib114] Heffernan S.M., Horner K., De Vito G., Conway G.E. (2019). The role of mineral and trace element supplementation in exercise and athletic performance: a systematic review. Nutrients.

[bib115] Heileson J., Sergi T., de Souza L.C. (2025). The omega-3 index in athletes: brief review. J. Exerc. Nutr..

[bib116] Herrera-Quintana L., Navajas-Porras B., Vázquez-Lorente H., Hinojosa-Nogueira D., Corrales-Borrego F.J., Lopez-Garzon M., Plaza-Diaz J. (2025). Celiac disease: beyond diet and food awareness. Foods.

[bib118] Hosseini S.M., Soltanizadeh N., Mirmoghtadaee P., Banavand P., Mirmoghtadaie L., Shojaee-Aliabadi S. (2018). Gluten-free products in celiac disease: nutritional and technological challenges and solutions. J. Res. Med. Sci..

[bib119] Hudson J.L., Bergia III R.E., Campbell W.W. (2020). Protein distribution and muscle-related outcomes: does the evidence support the concept?. Nutrients.

[bib120] Husby S., Murray J.A., Katzka D.A. (2019). AGA clinical practice update on diagnosis and monitoring of celiac disease—changing utility of serology and histologic measures: expert review. Gastroenterology.

[bib121] Hwang D.J., Yang H.J. (2024). Nutritional strategies for enhancing performance and training adaptation in weightlifters. Int. J. Mol. Sci..

[bib122] Infantino C., Francavilla R., Vella A., Cenni S., Principi N., Strisciuglio C., Esposito S. (2022). Role of vitamin D in celiac disease and inflammatory bowel diseases. Nutrients.

[bib123] Ip T.S.T., Fu S.C., Ong M.T.Y., Yung P.S.H. (2022). Vitamin D deficiency in athletes: laboratory, clinical and field integration. Asia-Pac. J. Sports Med. Arthrosc. Rehabil. Technol..

[bib125] Jäger R., Heileson J.L., Abou Sawan S., Dickerson B.L., Leonard M., Kreider R.B. (2025). International society of sports nutrition position stand: Long-chain omega-3 polyunsaturated fatty acids. J. Int. Soc. Sports Nutr..

[bib124] Jäger R., Kerksick C.M., Campbell B.I., Cribb P.J., Wells S.D., Skwiat T.M. (2017). International society of sports nutrition position stand: protein and exercise. J. Int. Soc. Sports Nutr..

[bib126] Jamieson J.A., Neufeld A. (2020). Food sources of energy and nutrients among Canadian adults following a gluten-free diet. PeerJ.

[bib127] Jedwab C.F., Roston B.C.D.M.B., Toge A.B.F.D.S., Echeverria I.F., Tavares G.O.G., Alvares M.A. (2022). The role of probiotics in the immune response and intestinal microbiota of children with celiac disease: a systematic review. Revista Paulista de Pediatria.

[bib128] Jimenez K., Kulnigg-Dabsch S., Gasche C. (2015). Management of iron deficiency anemia. Gastroenterol. Hepatol..

[bib129] Jivraj A., Hutchinson J.M., Ching E., Marwaha A., Verdu E.F., Armstrong D., Pinto-Sanchez M.I. (2022). Micronutrient deficiencies are frequent in adult patients with and without celiac disease on a gluten-free diet, regardless of duration and adherence to the diet. Nutrition.

[bib130] Joelson A.M., Choy A.M., Blackett J.W., Molinsky R.L., Geller M.G., Green P.H., Lebwohl B. (2021). Probiotic use in celiac disease: results from a national survey. J. Gastrointest. Liver Dis..

[bib131] Jung H.C., Seo M.W., Lee S., Kim S.W., Song J.K. (2018). Vitamin D3 supplementation reduces the symptoms of upper respiratory tract infection during winter training in vitamin D-insufficient taekwondo athletes: a randomized controlled trial. Int. J. Environ. Res. Publ. Health.

[bib132] Jurov I., Keay N., Spudić D., Rauter S. (2022). Inducing low energy availability in trained endurance male athletes results in poorer explosive power. Eur. J. Appl. Physiol..

[bib133] Kaliciak I., Drogowski K., Garczyk A., Kopeć S., Horwat P., Bogdański P. (2022). Influence of gluten-free diet on gut microbiota composition in patients with coeliac disease: a systematic review. Nutrients.

[bib134] Kardasis W., Naquin E.R., Garg R., Arun T., Gopianand J.S., Karmakar E., Gnana-Prakasam J.P. (2023). The IRONy in athletic performance. Nutrients.

[bib135] Kawashima I., Hiraiwa H., Ishizuka S., Kawai R., Hoshino Y., Kusaka Y., Tsukahara T. (2021). Comparison of vitamin D sufficiency between indoor and outdoor elite male collegiate athletes. Nagoya J. Med. Sci..

[bib136] Keller K., Friedrich O., Treiber J., Quermann A., Friedmann-Bette B. (2024). Iron deficiency in athletes: prevalence and impact on VO2 peak. Nutrition.

[bib137] Kerksick C.M., Kulovitz M., Bagchi D., Nair S., Sen C.K. (2013). Requirement of energy, carbohydrates, proteins and fats for athletes. Nutr. Enhanc. Sports Perform..

[bib139] Kim M.H., Kim H. (2017). The roles of glutamine in the intestine and its implication in intestinal diseases. Int. J. Mol. Sci..

[bib142] King A.J., Rowe J.T., Burke L.M. (2020). Carbohydrate hydrogel products do not improve performance or gastrointestinal distress during moderate-intensity endurance exercise. Int. J. Sport Nutr. Exerc. Metabol..

[bib141] King J.C., Brown K.H., Gibson R.S., Krebs N.F., Lowe N.M., Siekmann J.H., Raiten D.J. (2016). Biomarkers of Nutrition for Development (BOND)—Zinc review. J. Nutr..

[bib138] Kılıç M., Özpınar A., Serteser M., Kilercik M., Serdar M. (2022). The effect of reticulocyte hemoglobin content on the diagnosis of iron deficiency anemia: a meta-analysis study. J. Med. Biochem..

[bib143] Knechtle B., Jastrzębski Z., Hill L., Nikolaidis P.T. (2021). Vitamin D and stress fractures in sport: preventive and therapeutic measures—a narrative review. Medicina.

[bib144] Koopman R., Crombach N., Gijsen A.P., Walrand S., Fauquant J., Kies A.K. (2009). Ingestion of a protein hydrolysate is accompanied by an accelerated in vivo digestion and absorption rate when compared with its intact protein. Am. J. Clin. Nutr..

[bib145] Kreutz J.M., Adriaanse M.P., van Der Ploeg E.M., Vreugdenhil A.C. (2020). Narrative review: nutrient deficiencies in adults and children with treated and untreated celiac disease. Nutrients.

[bib146] Krzywański J., Mikulski T., Pokrywka A., Młyńczak M., Krysztofiak H., Frączek B., Ziemba A. (2020). Vitamin B12 status and optimal range for hemoglobin formation in elite athletes. Nutrients.

[bib147] Książek A., Zagrodna A., Słowińska-Lisowska M. (2019). Vitamin D, skeletal muscle function and athletic performance in athletes—A narrative review. Nutrients.

[bib148] Kuja-Halkola R., Lebwohl B., Halfvarson J., Wijmenga C., Magnusson P.K., Ludvigsson J.F. (2016). Heritability of non-HLA genetics in coeliac disease: a population-based study in 107 000 twins. Gut.

[bib149] Kurien M., Trott N., Sleet S., Sanders D.S. (2018). Prescribing gluten-free foods in general practice. Br. J. Gen. Pract..

[bib150] Kvamme J.M., Sørbye S., Florholmen J., Halstensen T.S. (2022). Population-based screening for celiac disease reveals that the majority of patients are undiagnosed and improve on a gluten-free diet. Sci. Rep..

[bib151] Łagowska K., Bajerska J. (2021). Probiotic supplementation and respiratory infection and immune function in athletes: systematic review and meta-analysis of randomized controlled trials. J. Athl. Train..

[bib152] Lambert K., Ficken C. (2016). Cost and affordability of a nutritionally balanced gluten‐free diet: is following a gluten‐free diet affordable?. Nutr. Diet..

[bib153] Lane D.J., Richardson D.R. (2014). The active role of vitamin C in mammalian iron metabolism: much more than just enhanced iron absorption. Free Radical Biol. Med..

[bib154] Larretxi I., Txurruka I., Navarro V., Lasa A., Bustamante M.Á., Fernández-Gil M.D.P. (2019). Micronutrient analysis of gluten-free products: their low content is not involved in gluten-free diet imbalance in a cohort of celiac children and adolescent. Foods.

[bib155] Larson-Meyer D.E., Woolf K., Burke L. (2018). Assessment of nutrient status in athletes and the need for supplementation. Int. J. Sport Nutr. Exerc. Metabol..

[bib156] Laterza L., Cremon C., Coppola G., Settanni C.R., Maresca R., Strazzeri M. (2025). Multistrain probiotics plus vitamin d improve gut barrier function and gut microbiota composition in irritable bowel syndrome without constipation: results from a double-blind, randomized, placebo-controlled trial. Nutrients.

[bib157] Lázár-Molnár E., Snyder M. (2018). The role of human leukocyte antigen in celiac disease diagnostics. Clin. Lab. Med..

[bib158] Lebovits J., Lee A.R. (2023). Micronutrient considerations for celiac disease. Practical Gastroenterol..

[bib159] Lebwohl B., Haggård L., Emilsson L., Söderling J., Roelstraete B., Butwicka A. (2021). Psychiatric disorders in patients with a diagnosis of celiac disease during childhood from 1973 to 2016. Clin. Gastroenterol. Hepatol..

[bib160] Lee A.R., Lebwohl B., Lebovits J., Wolf R.L., Ciaccio E.J., Green P.H. (2021). Factors associated with maladaptive eating behaviors, social anxiety, and quality of life in adults with celiac disease. Nutrients.

[bib161] Leffler D.A., Edwards-George J., Dennis M., Schuppan D., Cook F., Franko D.L. (2008). Factors that influence adherence to a gluten-free diet in adults with celiac disease. Dig. Dis. Sci..

[bib164] Leonard M.M., Camhi S., Huedo-Medina T.B., Fasano A. (2015). Celiac disease genomic, environmental, microbiome, and metabolomic (CDGEMM) study design: approach to the future of personalized prevention of celiac disease. Nutrients.

[bib163] Leonard M.M., Fasano A. (2019). Gluten and celiac disease risk: is it just a matter of quantity?. JAMA.

[bib165] Leone J.E., Gray K.A., Massie J.E., Rossi J.M. (2005). Celiac disease symptoms in a female collegiate tennis player: a case report. J. Athl. Train..

[bib166] Leone J.E., Wise K.A., Mullin E.M., Gray K.A., Szlosek P.A., Griffin M.F., Jordan C.A. (2020). Celiac disease symptoms in athletes: prevalence indicators of perceived quality of life. Sports Health.

[bib167] Lerner B.A., Vo L.T.P., Yates S., Rundle A.G., Green P.H., Lebwohl B. (2019). Detection of gluten in gluten-free labeled restaurant food: analysis of crowd-sourced data. Official journal of the American College of Gastroenterology| ACG.

[bib168] Leszczyńska J., Szczepankowska A.K., Majak I., Mańkowska D., Smolińska B., Ścieszka S. (2024). Reducing immunoreactivity of gluten peptides by probiotic lactic acid bacteria for dietary management of gluten-related diseases. Nutrients.

[bib169] Lionetti E., Catassi C. (2014). Co-localization of gluten consumption and HLA-DQ2 and-DQ8 genotypes, a clue to the history of celiac disease. Dig. Liver Dis..

[bib170] Lis D.M., Stellingwerff T., Shing C.M., Ahuja K.D., Fell J.W. (2015). Exploring the popularity, experiences, and beliefs surrounding gluten-free diets in nonceliac athletes. Int. J. Sport Nutr. Exerc. Metabol..

[bib171] Logue D.M., Madigan S.M., Melin A., Delahunt E., Heinen M., Donnell S.J.M., Corish C.A. (2020). Low energy availability in athletes 2020: an updated narrative review of prevalence, risk, within-day energy balance, knowledge, and impact on sports performance. Nutrients.

[bib172] Lucendo A.J., García-Manzanares A. (2013). Bone mineral density in adult coeliac disease: an updated review. Rev. Esp. Enferm. Dig..

[bib173] Ludvigsson J.F., Card T., Ciclitira P.J., Swift G.L., Nasr I., Sanders D.S., Ciacci C. (2015). Support for patients with celiac disease: a literature review. United Eur. Gastroenterol. J..

[bib174] Luettig J., Rosenthal R., Barmeyer C., Schulzke J.D. (2015). Claudin-2 as a mediator of leaky gut barrier during intestinal inflammation. Tissue Barriers.

[bib175] Lupu V.V., Trandafir L.M., Raileanu A.A., Mihai C.M., Morariu I.D., Starcea I.M. (2023). Advances in understanding the human gut microbiota and its implication in pediatric celiac disease—a narrative review. Nutrients.

[bib176] Lutsey P.L., Chen L.Y., Eaton A., Jaeb M., Rudser K.D., Neaton J.D., Alonso A. (2018). A pilot randomized trial of oral magnesium supplementation on supraventricular arrhythmias. Nutrients.

[bib177] Ma C., Singh S., Jairath V., Radulescu G., Ho S.K., Choi M.Y. (2022). Food insecurity negatively impacts gluten avoidance and nutritional intake in patients with celiac disease. J. Clin. Gastroenterol..

[bib178] Maggioni M.A., Rundfeldt L.C., Gunga H.C., Joerres M., Merati G., Steinach M. (2020). The advantage of supine and standing heart rate variability analysis to assess training status and performance in a walking ultramarathon. Front. Physiol..

[bib179] Makhlouf S., Messelmani M., Zaouali J., Mrissa R. (2018). Cognitive impairment in celiac disease and non-celiac gluten sensitivity: review of literature on the main cognitive impairments, the imaging and the effect of gluten free diet. Acta Neurol. Belg..

[bib180] Mancin L., Burke L.M., Rollo I. (2025). Fibre: the forgotten carbohydrate in sports nutrition recommendations. Sports Med..

[bib181] Mancini L.A., Trojian T., Mancini A.C. (2011). Celiac disease and the athlete. Curr. Sports Med. Rep..

[bib182] Marciniak M., Szymczak-Tomczak A., Mahadea D., Eder P., Dobrowolska A., Krela-Kaźmierczak I. (2021). Multidimensional disadvantages of a gluten-free diet in celiac disease: a narrative review. Nutrients.

[bib183] Martina S., Fabiola F., Federica G., Chiara B., Gioacchino L., Gian L.D.A. (2018). Genetic susceptibilty and celiac disease: what role do HLA haplotypes play?. Acta Biomed.: Atenei Parmensis.

[bib184] Martínez-Rodríguez A., Loaiza-Martínez D.A., Sánchez-Sánchez J., Rubio-Arias J.Á., Alacid F., Prats-Moya S. (2022). Personalised nutritional plan and resistance exercise program to improve health parameters in celiac women. Foods.

[bib185] Matera M., Guandalini S. (2024). How the microbiota may affect celiac disease and what we can do. Nutrients.

[bib186] Mathisen T.F., Ackland T., Burke L.M., Constantini N., Haudum J., Macnaughton L.S. (2023). Best practice recommendations for body composition considerations in sport to reduce health and performance risks: a critical review, original survey and expert opinion by a subgroup of the IOC consensus on Relative Energy Deficiency in Sport (REDs). Br. J. Sports Med..

[bib187] Mayes C., Meloni M. (2024). Forgetting how we ate: personalised nutrition and the strategic uses of history. Hist. Philos. Life Sci..

[bib188] McNally S.L., Donohue M.C., Newton K.P., Ogletree S.P., Conner K.K., Ingegneri S.E., Kagnoff M.F. (2012). Can consumers trust web-based information about celiac disease? Accuracy, comprehensiveness, transparency, and readability of information on the internet. Interactive journal of medical research.

[bib189] Melini V., Melini F. (2019). Gluten-free diet: gaps and needs for a healthier diet. Nutrients.

[bib190] Merkel D.L. (2013). Youth sport: positive and negative impact on young athletes. Open Access J. Sports Med..

[bib191] Messing B., Dutra S.L., Thuillier F., Darmaun D., Desjeux J.F. (1998). Whole-body protein metabolism assessed by leucine and glutamine kinetics in adult patients with active celiac disease. Metabolism.

[bib192] Micheletti A., Rossi R., Rufini S. (2001). Zinc status in athletes: relation to diet and exercise. Sports Med..

[bib193] Miranda J., Lasa A., Bustamante M.A., Churruca I., Simon E. (2014). Nutritional differences between a gluten-free diet and a diet containing equivalent products with gluten. Plant Foods Hum. Nutr..

[bib194] Moawad M.H.E., Alkhawaldeh I.M., Naswhan A.J. (2023). Efficacy of probiotics supplementation in amelioration of celiac disease symptoms and enhancement of immune system. World J. Clin. Cases.

[bib195] Moawad M.H.E., Serag I., Shalaby M.M., Aissani M.S., Sadeq M.A., Hendi N.I. (2024). Anxiety and depression among adults and children with celiac disease: a meta‐analysis of different psychiatry scales. Psychiatric Research and Clinical Practice.

[bib196] Montoro-Huguet M.A., Santolaria-Piedrafita S., Cañamares-Orbis P., García-Erce J.A. (2021). Iron deficiency in celiac disease: prevalence, health impact, and clinical management. Nutrients.

[bib197] Morenas-Aguilar M.D., Miras-Moreno S., Chacón-Ventura S., Martín-Olmedo J.J., Cwiklinska M., Jiménez-Martínez P. (2025). Highly branched cyclic dextrin supplementation and resistance training: a randomized double-blinded crossover trial examining mechanical, metabolic, and perceptual responses. Clinical Nutrition ESPEN.

[bib198] Morrell K., Melby M.K. (2017). Celiac disease: the evolutionary paradox. Int. J. Celiac Dis..

[bib199] Moss K., Kreutzer A., Graybeal A.J., Zhang Y., Braun-Trocchio R., Porter R.R., Shah M. (2023). Nutrient adequacy in endurance athletes. Int. J. Environ. Res. Publ. Health.

[bib200] Mozafarybazargany M., Khonsari M., Sokoty L., Ejtahed H.S., Qorbani M. (2023). The effects of probiotics on gastrointestinal symptoms and microbiota in patients with celiac disease: a systematic review and meta-analysis on clinical trials. Clin. Exp. Med..

[bib201] Muhammad H., Reeves S., Jeanes Y.M. (2019). Identifying and improving adherence to the gluten-free diet in people with coeliac disease. Proc. Nutr. Soc..

[bib202] Munn Z., Peters M.D., Stern C., Tufanaru C., McArthur A., Aromataris E. (2018). Systematic review or scoping review? Guidance for authors when choosing between a systematic or scoping review approach. BMC Med. Res. Methodol..

[bib203] Myint Z.W., Oo T.H., Thein K.Z., Tun A.M., Saeed H. (2018). Copper deficiency anemia. Ann. Hematol..

[bib204] Naderi A., Gobbi N., Ali A., Berjisian E., Hamidvand A., Forbes S.C. (2023). Carbohydrates and endurance exercise: a narrative review of a food first approach. Nutrients.

[bib205] Nakshabendi I.M., Downie S., Russell R.I., Rennie M.J. (1996). Increased rates of duodenal mucosal protein synthesis in vivo in patients with untreated coelia disease. Gut.

[bib206] Nikniaz Z., Beheshti S., Abbasalizad Farhangi M., Nikniaz L. (2021). A systematic review and meta‐analysis of the prevalence and odds of eating disorders in patients with celiac disease and vice‐versa. Int. J. Eat. Disord..

[bib207] Nikoo Goftar M., Sangani A., Jangi P. (2019). The effectiveness of cognitive-behavioral therapy based on mindfulness on anxiety, motivations and performance of track and field athletes. Organizational Behavior Management in Sport Studies.

[bib208] Nolte S., Krüger K., Hollander K., Carlsohn A. (2024). Approaches to prevent iron deficiency in athletes. Deutsche Zeitschrift fur Sportmedizin.

[bib209] Nunes C.L., Matias C.N., Santos D.A., Morgado J.P., Monteiro C.P., Sousa M. (2018). Characterization and comparison of nutritional intake between preparatory and competitive phase of highly trained athletes. Medicina.

[bib210] Nunes E.A., Colenso‐Semple L., McKellar S.R., Yau T., Ali M.U., Fitzpatrick‐Lewis D. (2022). Systematic review and meta‐analysis of protein intake to support muscle mass and function in healthy adults. J. Cachexia Sarcopenia Muscle.

[bib211] Ojetti V., Nucera G., Migneco A., Gabrielli M., Lauritano C., Danese S. (2005). High prevalence of celiac disease in patients with lactose intolerance. Digestion.

[bib212] Olshan K.L., Leonard M.M. (2023). Nutrition and risk of celiac disease–you are what you (wh) eat. Am. J. Clin. Nutr..

[bib213] Olsson C., Lyon P., Hörnell A., Ivarsson A., Sydner Y.M. (2009). Food that makes you different: the stigma experienced by adolescents with celiac disease. Qual. Health Res..

[bib214] Page M.J., McKenzie J.E., Bossuyt P.M., Boutron I., Hoffmann T.C., Mulrow C.D. (2021). The PRISMA 2020 statement: an updated guideline for reporting systematic reviews. Br. Med. J..

[bib215] Palsson O.S., Ballou S. (2020). Hypnosis and cognitive behavioral therapies for the management of gastrointestinal disorders. Curr. Gastroenterol. Rep..

[bib216] Pantaleoni S., Luchino M., Adriani A., Pellicano R., Stradella D., Ribaldone D.G. (2014). Bone mineral density at diagnosis of celiac disease and after 1 year of gluten‐free diet. Sci. World J..

[bib217] Peeling P., McKay A. (2023). Iron regulation and absorption in athletes: contemporary thinking and recommendations. Curr. Opin. Clin. Nutr. Metab. Care.

[bib218] Pes G.M., Bibbò S., Dore M.P. (2019). Coeliac disease: beyond genetic susceptibility and gluten. A narrative review. Ann. Med..

[bib219] Petterson H., Olson B.L. (2017). Effects of mindfulness-based interventions in high school and college athletes for reducing stress and injury, and improving quality of life. J. Sport Rehabil..

[bib220] Pettersson J., Hindorf U., Persson P., Bengtsson T., Malmqvist U., Werkström V., Ekelund M. (2008). Muscular exercise can cause highly pathological liver function tests in healthy men. Br. J. Clin. Pharmacol..

[bib221] Pikosky M.A., Cifelli C.J., Agarwal S., Fulgoni III V.L. (2022). Association of dietary protein intake and grip strength among adults aged 19+ years: NHANES 2011–2014 analysis. Front. Nutr..

[bib222] Pinto-Sanchez M.I., Bai J.C. (2019). Toward new paradigms in the follow up of adult patients with celiac disease on a gluten-free diet. Front. Nutr..

[bib223] Pitt A., Lerigo F., Satherley R.M. (2024). Health-related stigma and challenges faced by men living with celiac disease: a qualitative analysis. J. Acad. Nutr. Diet..

[bib224] Plotnikoff G.A. (2014). Time to reject the “One Size Fits All” myth. Glob. Adv. Health Med..

[bib225] Płudowski P., Kos-Kudła B., Walczak M., Fal A., Zozulińska-Ziółkiewicz D., Sieroszewski P. (2023). Guidelines for preventing and treating vitamin D deficiency: a 2023 update in Poland. Nutrients.

[bib226] Pulido O.M., Gillespie Z., Zarkadas M., Dubois S., Vavasour E., Rashid M. (2009). Introduction of oats in the diet of individuals with celiac disease: a systematic review. Adv. Food Nutr. Res..

[bib227] Qashqari L., Shakweer D., Alzaben A.S., Hanbazaza M.A. (2024). Investigation of cost and availability of gluten-free food in Jeddah, KSA. J. Taibah Univ. Med. Sci..

[bib228] Rabiee R., Mahdavi R., Nikniaz Z. (2024). Nutrition Clinique Et Métabolisme.

[bib229] Rađen S., Rađen G., Životić-Vanović M., Radaković S., Vasiljević N., Stojanović D. (2011). Effect of iron supplementation on maximal oxygen uptake in female athletes. Vojnosanit. Pregl..

[bib230] Ralphs D.E., Piper T.J. (2016). Celiac disease: a review for the athlete and interdisciplinary team. Strength Condit. J..

[bib231] Rana S.V., Malik A. (2014). Hydrogen breath tests in gastrointestinal diseases. Indian J. Clin. Biochem..

[bib232] Ravikumar N., Chegukrishnamurthi M., Gadde Venkata S. (2022). Role of Nutrients in Neurological Disorders.

[bib233] Ravisankar P., Reddy A.A., Nagalakshmi B., Koushik O.S., Kumar B.V., Anvith P.S. (2015). The comprehensive review on fat soluble vitamins. IOSR J. Pharm..

[bib234] Reilly N.R., Lebwohl B., Hultcrantz R., Green P.H., Ludvigsson J.F. (2015). Increased risk of non-alcoholic fatty liver disease after diagnosis of celiac disease. J. Hepatol..

[bib235] Ribichini E., Scalese G., Cesarini A., Mocci C., Pallotta N., Severi C., Corazziari E.S. (2023). Exercise-induced gastrointestinal symptoms in endurance sports: a review of pathophysiology, symptoms, and nutritional management. Dietetics.

[bib236] Rodrigo L. (2006). Celiac disease. World J. Gastroenterol.: WJG.

[bib237] Rogers D.L., Tanaka M.J., Cosgarea A.J., Ginsburg R.D., Dreher G.M. (2024). How mental health affects injury risk and outcomes in athletes. Sports Health.

[bib238] Roma E., Roubani A., Kolia E., Panayiotou J., Zellos A., Syriopoulou V.P. (2010). Dietary compliance and life style of children with coeliac disease. J. Hum. Nutr. Diet..

[bib239] Roman S., Campos-Medina L., Leal-Mercado L. (2024). Personalized nutrition: the end of the one-diet-fits-all era. Front. Nutr..

[bib241] Rondanelli M., Faliva M.A., Gasparri C., Peroni G., Naso M., Picciotto G. (2019). Micronutrients dietary supplementation advices for celiac patients on long-term gluten-free diet with good compliance: a review. Medicina.

[bib242] Rubio-Tapia A., Hill I.D., Semrad C., Kelly C.P., Greer K.B., Limketkai B.N., Lebwohl B. (2023). American college of gastroenterology guidelines update: diagnosis and management of celiac disease. Official journal of the American College of Gastroenterology| ACG.

[bib243] Sabzevari F., Samadi H., Ayatizadeh F., Machado S. (2023). Effectiveness of mindfulness-acceptance-commitment based approach for rumination, cognitive flexibility and sports performance of elite players of beach soccer: a randomized controlled trial with 2-months Follow-up. Clin. Pract. Epidemiol. Ment. Health: CP & EMH.

[bib244] Sacks F.M., Lichtenstein A.H., Wu J.H., Appel L.J., Creager M.A., Kris-Etherton P.M. (2017). Dietary fats and cardiovascular disease: a presidential advisory from the American Heart Association. Circulation.

[bib245] Sadowska-Krępa E., Rozpara M., Rzetecki A., Bańkowski S., Żebrowska A., Pilch W. (2021). Strenuous 12-h run elevates circulating biomarkers of oxidative stress, inflammation and intestinal permeability in middle-aged amateur runners: a preliminary study. PLoS One.

[bib246] Salazar C., García-Cárdenas J.M., Paz-y-Miño C. (2017). Understanding celiac disease from genetics to the future diagnostic strategies. Clin. Med. Insights Gastroenterol..

[bib247] Sallese M., Lopetuso L.R., Efthymakis K., Neri M. (2020). Beyond the HLA genes in gluten-related disorders. Front. Nutr..

[bib248] Samuels C., James L., Lawson D., Meeuwisse W. (2016). The athlete sleep screening questionnaire: a new tool for assessing and managing sleep in elite athletes. Br. J. Sports Med..

[bib249] Sanchis-Gomar F., Lippi G. (2014). Physical activity-an important preanalytical variable. Biochem. Med..

[bib250] Sandvik J., Potteiger K., Merlo A.R., Brody L.T. (2020). Celiac disease: university accommodations and support for student-athletes. Athl. Train. Sports Health Care.

[bib251] Sarita B., Samadhan D., Hassan M.Z., Kovaleva E.G. (2025). A comprehensive review of probiotics and human health-current prospective and applications. Front. Microbiol..

[bib252] Sarkhy A., El Mouzan M.I., Saeed E., Alanazi A., Alghamdi S., Anil S., Assiri A. (2016). Socioeconomic impacts of gluten-free diet among Saudi children with celiac disease. Pediatr. Gastroenterol. Hepatol. Nutr..

[bib253] Satherley R.M., Higgs S., Howard R. (2017). Disordered eating patterns in coeliac disease: a framework analysis. J. Hum. Nutr. Diet..

[bib254] Seiler C.L., Kiflen M., Stefanolo J.P., Bai J.C., Bercik P., Kelly C.P. (2020). Probiotics for celiac disease: a systematic review and meta-analysis of randomized controlled trials. Official journal of the American College of Gastroenterology| ACG.

[bib255] Sekiguchi Y., Giersch G.E., Jordan D.R., Dunn L., Fiol A., Lopez I.I.I.V. (2022). Countermovement jump, handgrip, and balance performance change during euhydration, mild-dehydration, rehydration, and ad libitum drinking. Journal of Exercise Science & Fitness.

[bib256] Seo D.I., Kim E., Fahs C.A., Rossow L., Young K., Ferguson S.L. (2012). Reliability of the one-repetition maximum test based on muscle group and gender. J. Sports Sci. Med..

[bib257] Serena G., Lima R., Fasano A. (2019). Genetic and environmental contributors for celiac disease. Curr. Allergy Asthma Rep..

[bib258] Sevinc E., Sevinc N., Akar H.H., Ozelcoskun B.D., Sezgin G.C., Arslan D., Kendirci M. (2016). Plasma glutamine and cystine are decreased and negatively correlated with endomysial antibody in children with celiac disease. Asia Pac. J. Clin. Nutr..

[bib259] Shariatpanahi Z.V., Eslamian G., Ardehali S.H., Baghestani A.R. (2019). Effects of early enteral glutamine supplementation on intestinal permeability in critically ill patients. Indian J. Crit. Care Med.: Peer-reviewed, Official Publication of Indian Society of Critical Care Medicine.

[bib260] Sharma A., Liu X., Hadley D., Hagopian W., Liu E., Chen W.M. (2016). Identification of non-HLA genes associated with celiac disease and country-specific differences in a large, international pediatric cohort. PLoS One.

[bib262] Shepherd J.A., Ng B.K., Sommer M.J., Heymsfield S.B. (2017). Body composition by DXA. Bone.

[bib261] Shepherd S.J., Gibson P.R. (2013). Nutritional inadequacies of the gluten‐free diet in both recently‐diagnosed and long‐term patients with coeliac disease. J. Hum. Nutr. Diet..

[bib263] Simón E., Molero-Luis M., Fueyo-Díaz R., Costas-Batlle C., Crespo-Escobar P., Montoro-Huguet M.A. (2023). The gluten-free diet for celiac disease: critical insights to better understand clinical outcomes. Nutrients.

[bib265] Singh P., Arora A., Strand T.A., Leffler D.A., Catassi C., Green P.H., Makharia G.K. (2018). Global prevalence of celiac disease: systematic review and meta-analysis. Clin. Gastroenterol. Hepatol..

[bib264] Singh P., Makharia G.K. (2014). Non-classical celiac disease: often missed. Int. J. Celiac Dis..

[bib267] Solberg A., Reikvam H. (2023). Iron status and physical performance in athletes. Life.

[bib268] Song Q.H., Xu R.M., Zhang Q.H., Shen G.Q., Ma M., Zhao X.P. (2015). Glutamine supplementation and immune function during heavy load training. Int. J. Clin. Pharm. Ther..

[bib269] Srivastava S., Tamrakar S., Nallathambi N., Vrindavanam S.A., Prasad R., Kothari R. (2024). Assessment of maximal oxygen uptake (VO2 Max) in athletes and Nonathletes assessed in sports physiology laboratory. Cureus.

[bib270] Standing Committee on the Scientific Evaluation of Dietary Reference Intakes, Subcommittee of Interpretation, Uses of Dietary Reference Intakes (2002). Dietary Reference Intakes for Vitamin A, Vitamin K, Arsenic, Boron, Chromium, Copper, Iodine, Iron, Manganese, Molybdenum, Nickel, Silicon, Vanadium, and Zinc.

[bib271] Staudacher H.M., Gibson P.R. (2015). How healthy is a gluten-free diet?. Br. J. Nutr..

[bib272] Stavitz J., Koc T. (2024). Exploring the experiences and perspectives of division III athletes regarding personalized nutrition plans for improved performance—A qualitative investigation. Healthcare.

[bib274] Stellingwerff T., Mountjoy M., McCluskey W.T., Ackerman K.E., Verhagen E., Heikura I.A. (2023). Review of the scientific rationale, development and validation of the International Olympic Committee Relative Energy Deficiency in Sport Clinical Assessment Tool: V. 2 (IOC REDs CAT2)—by a subgroup of the IOC consensus on REDs. Br. J. Sports Med..

[bib273] Stellingwerff T., Peeling P., Garvican-Lewis L.A., Hall R., Koivisto A.E., Heikura I.A., Burke L.M. (2019). Nutrition and altitude: strategies to enhance adaptation, improve performance and maintain health: a narrative review. Sports Med..

[bib275] Stroebele‐Benschop N., Rau C.J., Dieze A., Bschaden A. (2025). Life challenges and quality of life of people living with coeliac disease: time of diagnosis matters. J. Hum. Nutr. Diet..

[bib276] Sutehall S., Pitsiladis Y. (2025). Personalized nutrition for the enhancement of elite athletic performance. Scand. J. Med. Sci. Sports.

[bib277] Swaid F., Sukhotnik I., Matter I., Berkowitz D., Hadjittofi C., Pollak Y., Lavy A. (2013). Dietary glutamine supplementation prevents mucosal injury and modulates intestinal epithelial restitution following acetic acid induced intestinal injury in rats. Nutr. Metab..

[bib278] Talarico V., Giancotti L., Mazza G.A., Miniero R., Bertini M. (2021). Iron deficiency anemia in celiac disease. Nutrients.

[bib279] Tan I.L., Coutinho de Almeida R., Modderman R., Stachurska A., Dekens J., Barisani D. (2021). Circulating miRNAs as potential biomarkers for celiac disease development. Front. Immunol..

[bib280] Tansel A., Levinthal D.J. (2023). Understanding our tests: hydrogen-methane breath testing to diagnose small intestinal bacterial overgrowth. Clin. Transl. Gastroenterol..

[bib281] Tarsitano M.G., Quinzi F., Folino K., Greco F., Oranges F.P., Cerulli C., Emerenziani G.P. (2024). Effects of magnesium supplementation on muscle soreness in different type of physical activities: a systematic review. J. Transl. Med..

[bib282] Theethira T.G., Dennis M. (2015). Celiac disease and the gluten-free diet: consequences and recommendations for improvement. Dig. Dis..

[bib283] Thomas D.T., Erdman K.A., Burke L.M. (2016). Nutrition and athletic performance. Med. Sci. Sports Exerc..

[bib284] Thompson T., Dennis M., Higgins L.A., Lee A.R., Sharrett M.K. (2005). Gluten‐free diet survey: are Americans with coeliac disease consuming recommended amounts of fibre, iron, calcium and grain foods?. J. Hum. Nutr. Diet..

[bib285] Tomczyk M., Heileson J.L., Babiarz M., Calder P.C. (2023). Athletes can benefit from increased intake of EPA and DHA—Evaluating the evidence. Nutrients.

[bib286] Tomlinson P.B., Joseph C., Angioi M. (2015). Effects of vitamin D supplementation on upper and lower body muscle strength levels in healthy individuals. A systematic review with meta-analysis. J. Sci. Med. Sport.

[bib287] Vacca M., Porrelli A., Calabrese F.M., Lippolis T., Iacobellis I., Celano G. (2022). How metabolomics provides novel insights on celiac disease and gluten-free diet: a narrative review. Front. Microbiol..

[bib288] Vaishya R., Misra A., Vaish A., Ursino N., D'Ambrosi R. (2024). Hand grip strength as a proposed new vital sign of health: a narrative review of evidences. J. Health Popul. Nutr..

[bib289] Vereczkei Z., Farkas N., Hegyi P., Imrei M., Földi M., Szakács Z. (2021). It is high time for personalized dietary counseling in celiac disease: a systematic review and meta-analysis on body composition. Nutrients.

[bib290] Vici G., Belli L., Biondi M., Polzonetti V. (2016). Gluten free diet and nutrient deficiencies: a review. Clin. Nutr..

[bib291] Villanacci V., Del Sordo R., Lanzarotto F., Ricci C., Sidoni A., Manenti S. (2024). Claudin-2: a marker for a better evaluation of histological mucosal healing in inflammatory bowel diseases. Dig. Liver Dis..

[bib292] Wachsmuth N.B., Aberer F., Haupt S., Schierbauer J.R., Zimmer R.T., Eckstein M.L. (2022). The impact of a high-carbohydrate/low fat vs. low-carbohydrate diet on performance and body composition in physically active adults: a cross-over controlled trial. Nutrients.

[bib293] Walsh N.P. (2019). Nutrition and athlete immune health: new perspectives on an old paradigm. Sports Med..

[bib294] Wang F., Zheng J., Cheng J., Zou H., Li M., Deng B. (2022). Personalized nutrition: a review of genotype-based nutritional supplementation. Front. Nutr..

[bib295] Wang J., He Y., Liu Z., Liu X., Jing Y. (2025). Glutamine peptides: preparation, analysis, applications, and their role in intestinal barrier protection. Nutrients.

[bib296] Wasilewska M., Pietrzak K., Polok S., Bialic Ł. (2025). Iron deficiency in female endurance athletes: the role of hepcidin regulation, training load, and dietary strategies in optimizing performance and health. Quality in Sport.

[bib297] White L.E., Bannerman E., Gillett P.M. (2016). Coeliac disease and the gluten‐free diet: a review of the burdens; factors associated with adherence and impact on health‐related quality of life, with specific focus on adolescence. J. Hum. Nutr. Diet..

[bib298] Wieser H., Ruiz-Carnicer Á., Segura V., Comino I., Sousa C. (2021). Challenges of monitoring the gluten-free diet adherence in the management and follow-up of patients with celiac disease. Nutrients.

[bib299] Wieser H., Segura V., Ruiz-Carnicer Á., Sousa C., Comino I. (2021). Food safety and cross-contamination of gluten-free products: a narrative review. Nutrients.

[bib300] Williams P.M., Harris L.M., Odom M.R. (2022). Celiac disease: common questions and answers. Am. Fam. Physician.

[bib301] Witkowski M., Hubert J., Mazur A. (2012). Methods of assessment of magnesium status in humans: a systematic review. Magnes. Res..

[bib302] Wolf R.L., Lebwohl B., Lee A.R., Zybert P., Reilly N.R., Cadenhead J. (2018). Hypervigilance to a gluten-free diet and decreased quality of life in teenagers and adults with celiac disease. Dig. Dis. Sci..

[bib303] Woolf K., Manore M.M. (2006). B-vitamins and exercise: does exercise alter requirements?. Int. J. Sport Nutr. Exerc. Metabol..

[bib304] World Health Organization (2015). Guideline: Optimal Serum and Red Blood Cell Folate Concentrations in Women of Reproductive Age for Prevention of Neural Tube Defects.

[bib305] World Health Organization (2020).

[bib306] Wyatt P.B., Reiter C.R., Satalich J.R., O'Neill C.N., Edge C., Cyrus J.W. (2024). Effects of vitamin D supplementation in elite athletes: a systematic review. Orthop. J. Sports Med..

[bib307] Wynne B., McHugh L., Gao W., Keegan D., Byrne K., Rowan C. (2019). Acceptance and commitment therapy reduces psychological stress in patients with inflammatory bowel diseases. Gastroenterology.

[bib308] Yamanaka-Okumura H., Kawakami H., Oura M., Yamamoto S., Tsumura A., Miyazaki T. (2023). Effect of long-term intake of glycine and L-alanine powder at breakfast on skeletal muscle mass in older adults. Clinical Nutrition ESPEN.

[bib309] Yelland G.W. (2017). Gluten‐induced cognitive impairment (“brain fog”) in coeliac disease. J. Gastroenterol. Hepatol..

[bib310] Yoon S., Kwon O., Kim J. (2021). Vitamin D in athletes: focus on physical performance and musculoskeletal injuries. Physical Activity and Nutrition.

[bib312] Zhang H., Wang R., Guo S., Tian Q., Zhang S., Guo L. (2023). Lower serum magnesium concentration and higher 24-h urinary magnesium excretion despite higher dietary magnesium intake in athletes: a systematic review and meta-analysis. Food Sci. Hum. Wellness.

[bib311] Zhang Y., Xun P., Wang R., Mao L., He K. (2017). Can magnesium enhance exercise performance?. Nutrients.

[bib313] Zheng Y., Zhang Z., Tang P., Wu Y., Zhang A., Li D. (2023). Probiotics fortify intestinal barrier function: a systematic review and meta-analysis of randomized trials. Front. Immunol..

[bib314] Zhou Q., Verne M.L., Fields J.Z., Lefante J.J., Basra S., Salameh H., Verne G.N. (2019). Randomised placebo-controlled trial of dietary glutamine supplements for postinfectious irritable bowel syndrome. Gut.

