# Supplementary material for: An integrated nutritional and performance monitoring framework for athletes with celiac disease: A structured narrative review
Source: Curr Res Food Sci. 2026 Sep 22;13:101568. doi: 10.1016/j.crfs.2026.101568 (PMC13654455; doi:10.1016/j.crfs.2026.101568)
Supplement: Supplementary file 1 [file mmc1.docx]

**SANRA Checklist (Scale for the Assessment of Narrative Review Articles)**

**Manuscript title:** An Integrated Nutritional and Performance Monitoring Framework for Athletes with Celiac Disease: A Structured Narrative Review

| **#** | **SANRA Item** | **Justification / Location in Manuscript** |
| --- | --- | --- |
| 1 | **Justification of the article's importance for the readership** — Does the article explain why the topic is relevant, and to whom? | The Introduction and Abstract establish that CD is "an important yet under-researched condition among athletes," explicitly identify the target audience (clinicians, sports dietitians, coaches, athletic trainers, researchers), and state that "few existing reviews integrate nutritional management, nutritional supplementation, and functional performance monitoring within a single, athlete-centred review of celiac disease, a gap this review aims to address" (Introduction, final paragraph). |
| 2 | **Statement of concrete aims or formulation of questions** — Does the article contain an explicit statement of the aims of the review, ideally in the form of research questions? | The Abstract states the purpose explicitly: "to synthesize the current evidence on nutrient deficiencies, dietary management, and performance-monitoring strategies specific to athletes with CD, and to propose an integrated framework for their nutritional and functional assessment." Section 8 further restates the review's specific integrative aim relative to prior literature. |
| 3 | **Description of literature search** — Does the article describe when and how (which databases, search terms) the literature search was conducted? | Section 2.1 (Search Strategy) specifies the four databases searched (PubMed/MEDLINE, Cochrane, BioMed Central, BMJ collection), the search window (database inception through October 2025), the MeSH terms and free-text keywords used, the boolean combination logic, and the supplementary manual reference-list screening method. |
| 4 | **Referencing** — Is the referencing transparent, e.g., is a rationale given for the inclusion of certain types of evidence (and the exclusion of others), and is the quality of the evidence judged? | Section 2.2 provides explicit inclusion and exclusion criteria (peer-reviewed English-language articles; specific exclusion of editorials, opinion pieces, unpublished conference abstracts, single case reports without novel data, and non-English articles without translation). Section 2.3 describes a two-reviewer independent screening process with a defined evidence hierarchy (systematic reviews/RCTs/guidelines as highest evidence, observational studies as second-tier, other evidence as lowest tier), citing Munn et al. (2018). |
| 5 | **Scientific reasoning** — Does the article describe the state of scientific knowledge (e.g., accepted mechanisms of a disease) as it pertains to the review question, and does the line of arguments follow the standards of scientific reasoning, e.g., in evaluating cause-effect relationships? | Section 1 (Introduction) describes the accepted pathophysiological mechanism of CD (HLA-DQ2/DQ8-restricted T-cell activation, villous atrophy). Throughout Sections 4 to 7, mechanistic claims (e.g., exercise-induced intestinal permeability, hepcidin kinetics, claudin-2 upregulation) are consistently qualified by their level of evidence, with explicit caveats distinguishing animal/mechanistic data from human/athlete-specific data (e.g., Section 4.2: "it's important to note that these findings are based on animal studies and may not be directly translated to humans"). Section 2.3 explicitly states that conclusions are "narrative, integrative and hypothesis generating and are not to be interpreted as graded recommendations." |
| 6 | **Appropriate presentation of data** — Is the data presented appropriately, e.g., in terms of the reported subgroups or the endpoints chosen, and is the presentation of data (e.g., in figures or tables) helpful in terms of illustrating the main findings? | Table 1 synthesizes macronutrient, micronutrient, and supplement recommendations with corresponding challenges, intake strategies, dietary sources, and key references in a structured comparative format. Figure 1 illustrates villous atrophy relative to healthy mucosa with downstream systemic consequences. Figure 2 summarizes the nutritional, psychological, and socioeconomic challenges facing CD athletes in an integrated visual format. |
